# Increased peripheral inflammatory responses in myelin oligodendrocyte glycoprotein associated disease and aquaporin-4 antibody positive neuromyelitis optica spectrum disorder

**DOI:** 10.3389/fimmu.2022.1037812

**Published:** 2022-11-14

**Authors:** Angelika Bauer, Dagmar Rudzki, Klaus Berek, Alessandro Dinoto, Christian Lechner, Eva-Maria Wendel, Harald Hegen, Florian Deisenhammer, Thomas Berger, Romana Höftberger, Kevin Rostasy, Sara Mariotto, Markus Reindl

**Affiliations:** ^1^ Clinical Department of Neurology, Medical University of Innsbruck, Innsbruck, Austria; ^2^ VASCage Research Centre on Vascular Ageing and Stroke, Innsbruck, Austria; ^3^ Neurology Unit, Department of Neuroscience, Biomedicine, and Movement Sciences, University of Verona, Verona, Italy; ^4^ Department of Pediatrics I, Medical University of Innsbruck, Innsbruck, Austria; ^5^ Department of Neuropediatrics, Olgahospital/Klinikum Stuttgart, Stuttgart, Germany; ^6^ Department of Neurology, Medical University of Vienna, Vienna, Austria; ^7^ Comprehensive Center for Clinical Neurosciences and Mental Health, Medical University of Vienna, Vienna, Austria; ^8^ Division of Neuropathology and Neurochemistry, Department of Neurology, Medical University of Vienna, Vienna, Austria; ^9^ Paediatric Neurology, Witten/Herdecke University, Children’s Hospital Datteln, Datteln, Germany

**Keywords:** cytokines, chemokines, biomarkers, MOGAD, MOG-IgG, NMOSD, AQP4-IgG, MS

## Abstract

Autoantibody-associated demyelinating diseases of the central nervous system such as myelin oligodendrocyte glycoprotein-antibody associated disease (MOGAD) and aquaporin 4-antibody positive neuromyelitis optica spectrum disorders (AQP4+ NMOSD) are rare diseases but can cause severe disability. In both diseases, associated neuroinflammation is accompanied by blood and cerebrospinal fluid cytokine and chemokine signatures, which were shown to be distinct from those observed in patients with multiple sclerosis (MS). In this study, we aimed to confirm and extend these findings by analyzing a larger number of serum cytokines, chemokines and related molecules in patients with MOGAD or AQP4+ NMOSD in comparison to MS, to better understand the pathophysiology and to identify biomarkers potentially useful in clinical practice for diagnostic and treatment purposes. A total of 65 serum cytokines, chemokines and related molecules like growth factors and soluble receptors were measured by Procartaplex multiplex immunoassays in 40 MOGAD, 40 AQP4+ NMOSD and 54 MS patients at baseline. Furthermore, follow-up samples of 25 AQP4+ NMOSD and 40 MOGAD patients were measured after 6-12 months. Selected analytes were validated in a subgroup of samples using other bead-based assays and ELISA. At baseline, 36 analytes in MOGAD and 30 in AQP4+ NMOSD were significantly increased compared to MS. K-means cluster analysis of all significantly altered molecules revealed three distinct groups: Cluster I, including 12 MOGAD, 2 AQP4+ NMOSD and 3 MS patients, had a specific association with 11 IL-6/IL-17A associated cytokines. In this cluster, 9/17 (53%) patients were children. Cluster II with 13 MOGAD, 24 AQP4+ NMOSD and 1 MS patient was associated with 31 upregulated analytes. Cluster III contained 15 MOGAD, 14 AQP4+ NMOSD and 50 MS patients. In cluster II and III the majority were adults (82% and 92%). Most measured analytes remained stable over time. Validation of selected cytokines and chemokines using other analytical methods revealed moderate to high correlation coefficients, but absolute values differed between assays. In conclusion, these results obtained by bead-based multiplex assays highlight a significant association of biomarkers of peripheral inflammation in patients with antibody-associated demyelinating diseases in comparison with MS.

## 1 Introduction

Neuromyelitis optica (NMO) is a rare but devastating inflammatory demyelinating disorder of the central nervous system (CNS), in which highly specific antibodies were found targeting the water channel aquaporin-4 (AQP4+) on astrocytic endfeet (1). In 2015, NMO diagnostic criteria were revised to unify different NMO clinical presentations and the term NMO spectrum disorders (NMOSD) was established (2). In NMOSD, a relapsing disease course is common with predominantly bilateral optic nerve and longitudinally extensive spinal cord involvement, but also brainstem, diencephalon or hemispheric white matter lesions are typical (3).

Importantly, not all patients diagnosed with NMOSD are seropositive for antibodies directed against AQP4. In 50% of AQP4-IgG seronegative patients, antibodies targeting the myelin oligodendrocyte glycoprotein (MOG-IgG), which is mainly expressed on oligodendrocytes, can be found (4). However, MOG-IgG are also found in other CNS demyelinating syndromes such as unilateral or bilateral optic neuritis, myelitis or acute disseminated encephalomyelitis (ADEM) (4–7). The establishment of highly specific assays for testing MOG-IgG has led to the development of specific diagnostic criteria of MOG-IgG associated diseases (MOGAD) and, thus, MOGAD was classified as a separate CNS inflammatory demyelinating disease distinct from AQP4+ NMOSD and MS. Importantly, the clinical phenotype of MOGAD changes with age, with ADEM being more common in children and opticospinal manifestations being more common with increasing age. Apart from typical clinical presentation, MOG-IgG positivity needs to be shown to fulfill the MOGAD diagnostic criteria (7).

Since inflammation is a dominant factor in NMOSD, MOGAD or MS, neuroinflammation measured by cytokine and chemokine profiles in either blood or cerebrospinal fluid (CSF) has been a target of extensive research over the last years (8–10). In agreement with the distinct clinical, neuropathological, and immunological findings observed in NMOSD and MOGAD in comparison with MS (2, 4, 7), also cytokine and chemokine signatures were shown to differ (9, 10). Earlier studies have identified T-helper 17 (Th17; interleukin (IL)-6 and IL-17 associated) and Th2 (IL-4, IL-5, and IL-13 associated) dominant cytokine profiles in AQP4+ NMOSD and MOGAD patients (9–11), mainly in CSF, but also in serum. Among these cytokines, especially IL-6 was of particular interest, as it promotes Th17 cell differentiation (12), the production of AQP4-targeting antibodies in NMOSD by plasmablasts (13) and, thus, increases blood-brain barrier permeability and facilitates CNS inflammation (14). Strikingly, satralizumab, a humanized monoclonal recycling antibody binding to soluble and membrane-bound IL-6 receptors, inhibiting IL-6 binding and consequently IL-6 signaling pathways, was found to be clinically effective in two pivotal Phase 3 trials in NMOSD, especially in the AQP4+ NMOSD patient population (15, 16). Similarly, tocilizumab, another humanized anti-IL-6 receptor antibody, effectively reduced median annual relapse rate in a case series of AQP4+ NMOSD but also in MOGAD patients (17).

Therefore, the aim of this study was to extend serum profiles of cytokines, chemokines and related molecules in patients with MOGAD or AQP4+ NMOSD, with a particular focus on IL-6, to better understand underlying disease pathophysiology and find potential biomarkers to distinguish those antibody-mediated conditions from MS. As there is an urgent need for easily measurable, reproducible biomarkers to support diagnostic and treatment decisions, we chose to study cytokine, chemokine and related molecules in serum samples rather than in CSF.

## 2 Materials and methods

### 2.1 Samples and clinical data

After blood collection, blood samples were left at room temperature for 30 minutes (min) to allow clotting. Then they were centrifuged at 2,000 x g for 10 min and serum was immediately transferred into clean polypropylene tubes and stored at -80°C until use at three diagnostic centers (Medical University of Innsbruck, Austria; University Hospital of Verona, Italy; Medical University of Vienna, Austria) between 2012-2021. For shipment of serum samples between diagnostic centers, dry ice was used. Serum samples were included from 40 MOGAD, 40 AQP4+ NMOSD and 54 MS patients. Unfortunately, clinical information was not fully available for many patients. At the time of sample collection, which was either routinely or because of the occurrence of a clinical relapse, 3 out of 23 AQP4+ NMOSD patients, 9 out of 18 MOGAD patients, and 49 out of 54 people with MS were treatment-naïve. In the MOGAD cohort, especially children were among the treatment-naïve. Others received several different treatments since disease onset and during disease history. During a period of 30 days before blood was collected for this study, 7 out of 23 AQP4+ NMOSD patients and 10 out of 19 MOGAD patients, received an immunosuppressive therapy. Follow-up samples of 25 AQP4+ NMOSD and 40 MOGAD patients were taken after 6-12 months. Importantly, we only included samples from patients in whom blood collection for research purposes was performed before acute treatment was given such as steroid infusion or plasma exchange. Demographic and clinical data of patients are shown in **Table 1**.

**Table 1:** Demographic baseline characteristics of study participants.

	NMOSD	MOGAD	MS
Number of patients	40	40	54
F:M, n (%)	32 (80.0): 8 (20.0)	22 (55.0): 18 (45.0)	35 (64.8): 19 (35.2)
Median age (min-max), y	51.8 (3.5-86.2)	23.7 (3.3-72.0)	31.2 (19.5-58.3)
Participants <18 y	5 (12.5)	17 (42.5)	0 (0.0)
Median disease duration (min-max), mo	59.0 (0.0-373.0)	12.0 (0.0-202.0)	0.0 (0.0-173.0)
Monophasic disease course, n (%)	4/23 (17.4)	16/32 (50.0)	n.a.
Sample taking during relapse, n (%)	7/23 (30.4)	12/32 (37.5)	n.a.
Sample taking during remission, n (%)	16/23 (69.6)	20/32 (62.5)	n.a.
Immunosuppressive treatment in the last 30d, n (%)	7/23 (30.4) (2 PRED, 2 AZA, 1 MMF, 1 TOC, 1 RTX)	10/19 (52.6) (8 IVIG, 2 RTX)	0/54
Long-term treatment since disease onset, n (%)	20/23 (87.0) (*: 16 RTX, 7 AZA, 7 PRED, 3 TOC, 2 CYC, 2 MMF, 2 PLEX, 1 MTX, 1 IVIG, 1 Tx not further described)	9/18 (50.0) (6 IVIG, 3 RTX)	5/54 (9.3) (2 S1P receptor modulator, 3 DMF)
Median antibody titer at baseline (min-max) [available from n (%) patients]	640 (20-20,480) [25 (62.5)]	2050 (320-20,480) [40 (100%)]	
Number of patients with follow-up samples available	25	40	n.a.
Median time until follow-up sample (min-max), mo	6.0 (4.0-24.0)	12.0 (6.0-38.0)	
EDSS at sample collection, median (min-max)			1.0 (0.0-6.0)
MS subtype at sample collection			CIS:3 RRMS: 46 SPMS: 1 PPMS: 4

AZA, azathioprine; CIS, clinically isolated syndrome; CYC, cyclophosphamide; d,days; DMF, dimethyl fumarat; EDSS, expanded disability status scale; F, female; IVIG, intravenous immunoglobulin; M, male; mo, months; MMF, mycophenolate mofetil; MOGAD, myelin oligodendrocyte glycoprotein associated disease; MS, multiple sclerosis; MTX, methotrexate; n.a., not analyzed/data not available; NMOSD, neuromyelitis optica spectrum disorder; PLEX, plasma exchange; PRED, prednisone; PPMS, primary progressive multiple sclerosis; RRMS, relapsing remitting multiple sclerosis; RTX, rituximab; S1P, sphingosine-1-phosphate; SD, standard deviation; SPMS, secondary progressive multiple sclerosis; TOC, tocilizumab; Tx, treatment; y, years;*some patients received multiple different treatments over time.

The present study was approved by the ethical committees of the Medical University of Innsbruck, Austria (AM3041A and AM4059), Medical University of Vienna (EK 1636/2019 and 1123/2015) and 23 samples were obtained from the Neuropathology-Verona biobank. All patients or their legal representatives gave written informed consent to diagnostic procedures and biological sample storage for research purposes.

### 2.2 AQP4-IgG and MOG-IgG detection assays

Cell-based assays detecting IgG targeting AQP4 or MOG were performed using HEK293A cells transfected with full-length human AQP4 (isoform M23) or MOG, as previously described (18, 19).

### 2.3 65-Multiplex cytokine and chemokine assays

Levels of serum cytokines, chemokines and related molecules were measured using commercially available bead-based immunoassays (Thermo Fisher Scientific, Waltham, MA, USA; Immune monitoring 65-plex human ProcartaPlex panel, cat. #EPX650-10065-901), which can measure the following 65 cytokines, chemokines and related molecules simultaneously:

Cytokines: granulocyte colony-stimulating factor (G-CSF or CSF-3), granulocyte macrophage colony-stimulating factor (GM-CSF), Interferon (IFN)-α, IFN-γ, interleukin (IL)-1α, IL-1β, IL-2, IL-3, IL-4, IL-5, IL-6, IL-7, IL-8 (or CXCL8), IL-9, IL-10, IL-12p70, IL-13, IL-15, IL-16, IL-17A (or cytotoxic T-lymphocyte-associated protein (CTLA)-8), IL-18, IL-20, IL-21, IL-22, IL-23, IL-27, IL-31, Leukemia inhibitory factor (LIF), macrophage colony-stimulating factor (M-CSF), macrophage migration inhibitory factor (MIF), tumor necrosis factor (TNF)-α, TNF-β, thymic stromal lymphopoietin (TSLP)

Chemokines: B lymphocyte chemoattractant (BLC or CXC-chemokine ligand (CXCL)-CXCL13)), epithelial-derived neutrophil-activating peptide 78 (ENA-78 or CXCL5), Eotaxin (or CC-chemokine ligand (CCL)-11), Eotaxin-2 (or CC-chemokine ligand (CCL)-24), Eotaxin-3 (or CC-chemokine ligand (CCL)-26), Fractalkine (or CX3C-chemokine ligand (CX3CL)-1), growth-regulated oncogene (GRO)-α (or KC/CXCL1), IFN-'Y-induced protein (IP)-10 (or CXCL10), Interferon-inducible T-cell alpha chemoattractant (I-TAC or CXCL11), monocyte chemoattractant protein (MCP)-1 (or CCL2), MCP-2 (CCL8), MCP-3 (CCL7), Macrophage-derived chemokine (MDC or CCL22), monokine induced by interferon-γ (MIG or CXCL9), macrophage inflammatory protein ((MIP)-1α or CCL3), MIP-1β (CCL4), MIP-3α (CCL20), stromal cell-derived factor (SDF)-1α (or CXCL12)

Growth factors/regulators: fibroblast growth factor (FGF)-2, hepatocyte growth factor (HGF), Matrix metalloproteinase-1 (MMP-1), nerve growth factor (NGF)-β, stem-cell factor (SCF), Vascular endothelial growth factor (VEGF)-A

Soluble receptors: a proliferation-inducing ligand (APRIL), B cell activating factor (BAFF), CD30, CD40 ligand (CD40L (CD154)), IL-2R (CD25), TNFR2, Tumor necrosis factor-related apoptosis-inducing ligand (TRAIL or CD253), Tumor necrosis factor-like weak inducer of apoptosis (TWEAK).

Multiplex assays were performed according to the manufacturer’s instructions. Serum samples which were frozen at -80°C were thawed on ice and then used immediately. Briefly, magnetic beads were added into each well of a 96-well flat bottom plate. After washing the beads, 25µl of universal assay buffer was added, followed by 25µl of undiluted serum samples or four-fold serial diluted standards. After incubation for 120min at room temperature on a shaker at 500 rounds per minute (rpm), beads were washed and 25µl of detection antibody mixture was added. Next, after 30min incubation at room temperature on a shaker, beads were washed twice and 50µl of streptavidin-phycoerythrin solution was added to each well. Incubation of 30min whilst shaking at room temperature followed. After washing, 120µl of reading buffer was added into each well and incubated for 5min at room temperature with agitation at 500rpm. Finally, fluorescence intensity was measured using Luminex MAGPIX instrument (Software: xPonent 4.2 and ProcartaPlex Analyst 1.0) and the cytokine/chemokine concentrations were calculated using the standard curve generated by five-parameter logistic regression method. In samples where cytokines, chemokines and related molecules were undetectable, the values of the lower standard concentrations were used for analyses. For samples with concentrations above the highest standard, the concentration of the top standard was used for further analyses. Results obtained by this assay are termed as bead-based assay 1.

### 2.4 Validation of IL-6 levels

We were especially interested in IL-6 levels in our study cohort, as IL-6 targeting therapy satralizumab was already approved for AQP4+ NMOSD (15, 16). Therefore, the concentration of IL-6 in the serum was quantified by ELISA from five different manufacturers (Thermo Fisher Scientific, Waltham, MA, USA; IL-6 Human high sensitivity ELISA, cat. #BMS213HS, henceforth referred to as ELISA 1; R&D Systems, Minneapolis, MN, USA; Human IL-6 Quantikine ELISA, cat. #D6050, henceforth referred to as ELISA 2; Mabtech, Cincinnati, OH, USA; Human IL-6 ELISA Pro, cat. #3460-1HP-2, henceforth referred to as ELISA 3; Abcam, Cambridge, CB, UK; Human IL-6 ELISA Kit, cat. #ab178013, henceforth referred to as ELISA 4; Biolegend, San Diego, CA, USA; MAX Human IL-6 ELISA, cat. #430501, henceforth referred to as ELISA 5) according to the manufacturer’s instructions. Results were compared to bead-based assay 1 and with other bead-based assays described below. In samples where cytokines, chemokines and related molecules were undetectable, the values of the lower standard concentrations were used for analyses.

### 2.5 Validation of other cytokines, chemokines and related molecules

Levels of 18 selected cytokines, chemokines and related molecules were measured in a subgroup of samples on commercially available multi-analyte flow assay kits, namely RayPlex (RayBiotech, Peachtree Corners, GA, USA; Human inflammation array 1 (13-plex), cat #FAH-INF-1-50: G-CSF, IL-13, IL-2, IL-23p19, IL-4, IL-1β, IFN-γ, TNF-α, MCP-1, IL-6, IL-12p70, IL-17A, henceforth referred to as bead-based assay 2) and LegendPlex (Biolegend, San Diego, CA, USA; Human inflammation panel 1 (13-plex), cat #740809: IL-1b, IFNα2, IFN-γ, TNF-α, MCP-1, IL-6, IL-8, IL-10, IL-12p70, IL-17A, IL-18, IL-23, IL-33, henceforth referred to as bead-based assay 3). Additionally, Procartaplex human basic kits (Thermo Fisher Scientific, Waltham, MA, USA; cat. # EPX010-10420-901, henceforth referred to as bead-based assay 4) were combined to validate the levels of seven cytokines/chemokines, namely BLC, IL-6, IL-8, IL-16, IL-17A, IP-10, MDC. Assays were performed according to the manufacturer’s instructions.

Furthermore, the concentration of SDF-1α was analyzed using three different ELISA kits (Thermo Fisher Scientific, Waltham, MA, USA; SDF-1 alpha/CDCL12A human ELISA kit, cat. #EHCXCL12A, henceforth denoted as ELISA 6; R&D Systems, Minneapolis, MN, USA; Human CXCL12/SDF-1 alpha Quantikine ELISA, cat. #DSA00, henceforth denoted as ELISA 7; Abcam, Cambridge, CB, UK; Human SDF1 alpha ELISA Kit, cat. #ab100637, henceforth denoted as ELISA 8). In samples where cytokines, chemokines and related molecules were undetectable, the values of the lower standard concentrations were used for analyses.

### 2.6 Statistical analyses

Statistical analyses were performed using GraphPad Prism 9 (GraphPad Software, La Jolla, California, USA) and IBM SPSS software (IBM Corp. Released 2012. IBM SPSS Statistics; Version 27.0. Armonk, New York, USA: IBM Corp.). Data was log-transformed to reduce the influence of outliers. Two-group comparisons were done using Mann Whitney U test with two-stage step-up (Benjamini, Krieger and Yekutieli) and a false discovery rate (FDR) of 1%. p-values <0.05 were classified as significant. Comparisons between multiple groups were calculated using the Kruskal Wallis test with Dunn’s multiple comparison test. For cytokine, chemokine changes over time within the same patient, Wilcoxon test with Bonferroni-Dunn correction was used. K-means cluster analysis was performed to classify NMOSD, MOGAD and MS patients according to cytokine levels. To assess inter-assay comparability using different ELISA and various bead-based assays, again log-transformed data was used and Pearson’s correlation coefficients were calculated. We classified correlation coefficients between 0.90-1.00 as very strong, between 0.70-0.89 as strong, between 0.40-0.69 as moderate, between 0.10-0.39 as weak and 0.10 as negligible correlation (20).

## 3 Results

### 3.1 Significantly altered cytokines, chemokines and related molecules in serum samples from patients with neuroinflammatory disorders

**Figure 1:**
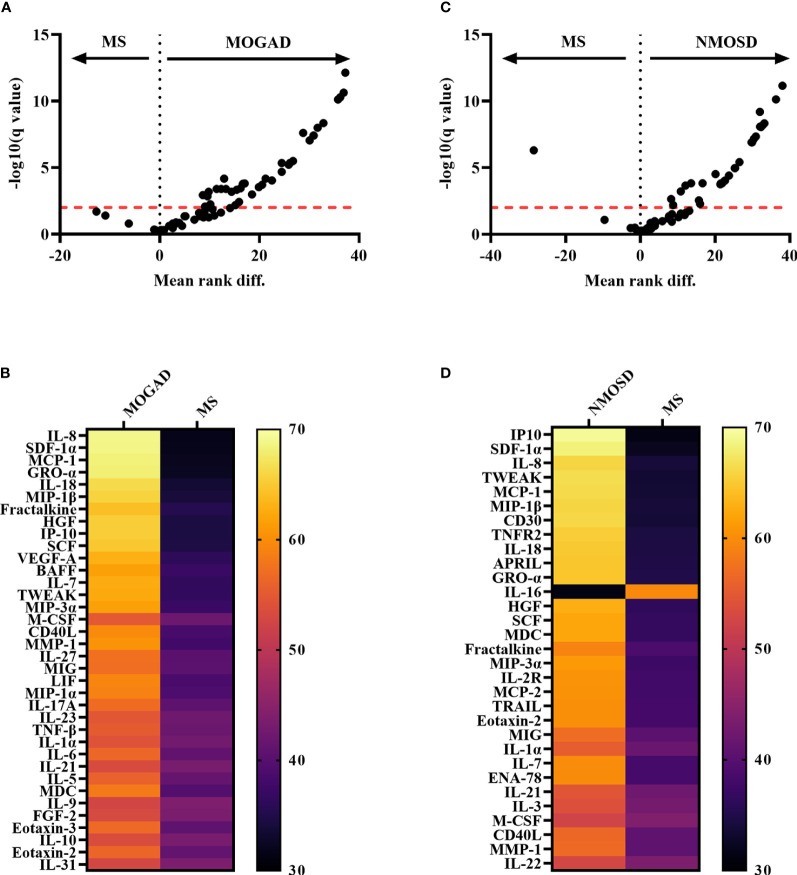
Comparison between autoantibody-associated diseases vs MS showing significantly altered cytokines, chemokines and related molecules. **(A)** Volcano Plot of Mann-Whitney tests after two-stage step-up (Benjamini, Krieger and Yekutieli) and FDR of 1% correction for multiple comparisons showing comparison of normalized levels (log10-transformation) of 65 cytokines, chemokines and related molecules (bead-based assay 1) between MOGAD and MS patients. The red dotted line indicates the level of significance after correction for multiple comparisons. Analytes above the red line are classified as significantly different between MOGAD and MS. **(B)** Heatmap showing mean ranks of significantly different cytokines, chemokines and related molecules comparing MOGAD and MS patients. **(C)** Volcano Plot of Mann-Whitney tests of 65 cytokines, chemokines and related molecules comparing NMOSD with MS patients after correction for multiple comparisons. Analytes above the red dotted line (level of significance after correction for multiple comparisons) are classified as statistically significant different between the two diseases. **(D)** Heatmap showing mean ranks of significantly different cytokines, chemokines and related molecules comparing NMOSD patients with MS patients. APRIL, a proliferation-inducing ligand; BAFF, B cell activation factor; BLC, B lymphocyte chemoattractant; CD40L, CD40 ligand; ENA-78, epithelial neutrophil-activating peptide-78; FGF, fibroblast growth factor; G-CSF, granulocyte colony-stimulating factor; GM-CSF, granulocyte-macrophage colony-stimulating factor; GRO, growth-regulated oncogene; HGF, hepatocyte growth factor; IFN, interferon; IL, interleukin; IP, interferon-ɣ-induced protein; I-TAC, interferon-inducible T cell α-chemoattractant; LIF, leukemia inhibitory factor; MCP, monocyte chemoattractant protein; M-CSF, macrophage colony-stimulating factor; MDC, macrophage-derived chemokine; MIF, macrophage migration inhibitory factor; MIG, monokine induced by interferon-ɣ; MIP, macrophage inflammatory protein; MMP, matrix metalloproteinase; MOGAD, myelin oligodendrocyte glycoprotein associated disease; MS, multiple sclerosis; NGF, nerve growth factor; NMOSD, neuromyelitis optica spectrum disorder; SCF, stem cell factor; SDF, stromal cell-derived factor; TNF, tumor necrosis factor; TRAIL, TNF-related apoptosis-inducing ligand; TSLP, thymic stromal lymphopoietin; TWEAK, tumor necrosis factor-like weak inducer of apoptosis; VEGF, vascular endothelial growth factor.

We analyzed levels of 65 cytokines, chemokines and related molecules in baseline serum samples and compared autoantibody-associated diseases with MS. Compared with MS, the levels of 36 analytes were significantly increased in MOGAD patients, which are namely IL-8, SDF-1α, MCP-1, GRO-α, IL-18, MIP-1β, Fractalkine, HGF, IP-10, SCF, VEGF-A, BAFF, IL-7, TWEAK, MIP-3α, M-CSF, CD40L, MMP-1, IL-27, MIG, LIF, MIP-1α, IL-17A, IL-23, TNF-β, IL-1α, IL-6, IL-21, IL-5, MDC, IL-9, FGF-2, Eotaxin-3, IL-10, Eotaxin-2, IL-31 (**Figures 1A, B**; **Supplementary Figure 1**).

In the AQP4+ NMOSD cohort compared to MS patients, 31 analytes were significantly different compared to MS: IP-10, SDF-1α, IL-8, TWEAK, MCP-1, MIP-1β, CD30, TNFR2, IL-18, APRIL, GRO-α, IL-16, HGF, SCF, MDC, Fractalkine, MIP-3α, IL-2R, MCP-2, TRAIL, Eotaxin-2, MIG, IL-1α, IL-7, ENA-78, IL-21, IL-3, M-CSF, CD40L, MMP-1, IL-22, with all being higher in AQP4+ NMOSD, except for IL-16 (**Figures 1C, D**; **Supplementary Figure 1**).

**Figure 2:**
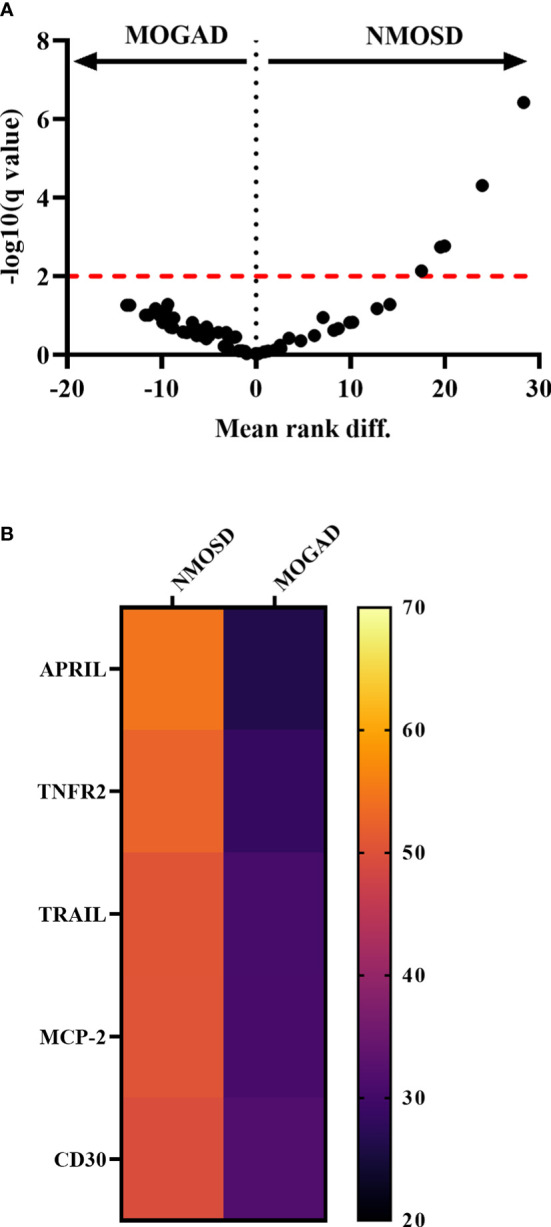
Comparison of cytokine, chemokine and related molecule levels between autoantibody-associated diseases. **(A)** Volcano Plot comparing MOGAD with NMOSD of all 65 cytokines, chemokines and related molecules measured by bead-based assay 1. Significant hits are shown above the significant level after correction for multiple comparisons using two-stage step-up (Benjamini, Krieger and Yekutieli) and FDR of 1% correction indicated as a red dotted line. **(B)** Heatmap showing mean ranks of significantly different cytokines, chemokines comparing MOGAD with NMOSD. APRIL, a proliferation-inducing ligand; BAFF, B cell activation factor; MCP, monocyte chemoattractant protein; MOGAD, myelin oligodendrocyte glycoprotein associated disease; MS, multiple sclerosis; NMOSD, neuromyelitis optica spectrum disorder; TNF, tumor necrosis factor; TRAIL, TNF-related apoptosis-inducing ligand.

A direct comparison of AQP4+ NMOSD with MOGAD revealed five analytes, which were significantly higher concentrated in NMOSD patients (APRIL, TNFR2, TRAIL, MCP-2, CD30; **Figure 2**; **Supplementary Figure 1**).

Comparing levels of cytokines, chemokines and related molecules within MOGAD and AQP4+ NMOSD patients revealed no significant differences between samples taken during relapse or remission, between monophasic or relapsing disease course and neither between those who received treatment or not. Furthermore, no difference was found between sex groups, children (i.e. younger than 18 years) and adults and between those who had at least two relapses compared to those with more relapses in both MOGAD or AQP4+ NMOSD groups (**Supplementary Figure 2**).

### 3.2 Association of clusters with distinct cytokines and chemokines

**Figure 3:**
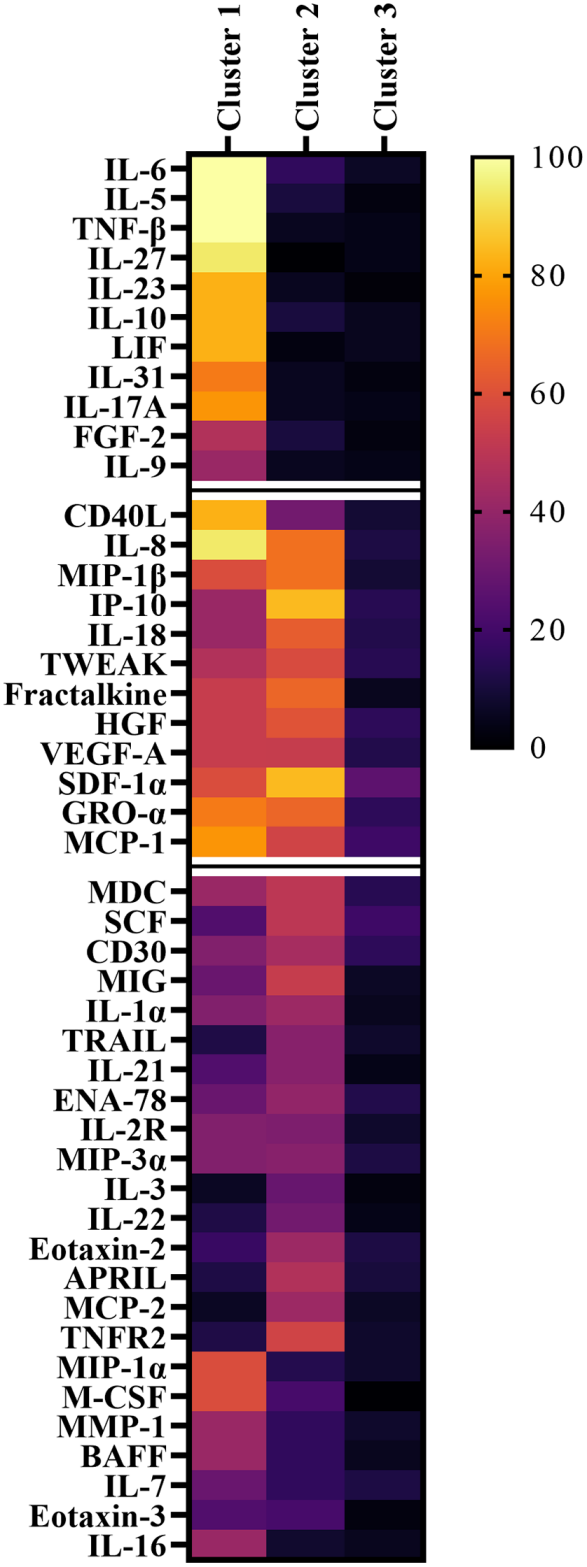
Heatmap of significantly altered cytokines, chemokines and related molecules showing the frequency of samples above the 90% specificity cut-off values for MS. Samples are classified in cluster 1 to 3 by k-means cluster analysis. Cytokines and chemokines are organized according to their frequency within the clusters. APRIL, a proliferation-inducing ligand; BAFF, B cell activation factor; BLC, B lymphocyte chemoattractant; CD40L, CD40 ligand; ENA-78, epithelial neutrophil-activating peptide-78; FGF, fibroblast growth factor; G-CSF, granulocyte colony-stimulating factor; GM-CSF, granulocyte-macrophage colony-stimulating factor; GRO, growth-regulated oncogene; HGF, hepatocyte growth factor; IFN, interferon; IL, interleukin; IP, interferon-ɣ-induced protein; I-TAC, interferon-inducible T cell α-chemoattractant; LIF, leukemia inhibitory factor; MCP, monocyte chemoattractant protein; M-CSF, macrophage colony-stimulating factor; MDC, macrophage-derived chemokine; MIF, macrophage migration inhibitory factor; MIG, monokine induced by interferon-ɣ; MIP, macrophage inflammatory protein; MMP, matrix metalloproteinase; MOGAD, myelin oligodendrocyte glycoprotein associated disease; MS, multiple sclerosis; NGF, nerve growth factor; NMOSD, neuromyelitis optica spectrum disorder; SCF, stem cell factor; SDF, stromal cell-derived factor; TNF, tumor necrosis factor; TRAIL, TNF-related apoptosis-inducing ligand; TSLP, thymic stromal lymphopoietin; TWEAK, tumor necrosis factor-like weak inducer of apoptosis; VEGF, vascular endothelial growth factor.

In a next step, cut-off values of all significantly dysregulated cytokines/chemokines were calculated to discriminate MOGAD and AQP4+ NMOSD from MS patients with 90% specificity (**Supplementary Table 1**). K-means cluster analysis was performed and showed three distinct groups (**Table 2**, **Figure 3**): Cluster I included 17 patients (12 MOGAD, 2 AQP4+ NMOSD and 3 MS), of whom 9 (52.9%) were aged younger than 18 years. Cluster II consisted of 38 patients (13 MOGAD, 24 AQP4+ NMOSD and 1 MS) with 81.6% being adults. Cluster III contained 79 patients (15 MOGAD, 14 AQP4+ NMOSD and 50 MS); the majority were adults (92.4%).

**Table 2:** Describing characteristics of clusters, distinguished by similar cytokine/chemokine profiles.

	Cluster 1	Cluster 2	Cluster 3
	n (%)	n (%)	n (%)
Number of cases	17 (12.7)	38 (28.4)	79 (59.0)
Diagnosis:			
MS	3 (17.6)	1 (2.6)	50 (63.3)
NMOSD	2 (11.8)	24 (63.2)**	14 (17.7)
MOGAD	12 (70.6)*	13 (34.2)**	15 (19.0)
Age:			
Children (<18)	9 (52.9)*	7 (18.4)	6 (7.6)
Adults (>18)	8 (47.1)	31 (81.6)	73 (92.4)
Sex:			
Female	12 (70.6)	27 (71.1)	50 (63.3)
Male	5 (29.4)	11 (28.9)	29 (36.7)
Disease status:			
Relapse	4/12 (33.3)	11/27 (40.7)	4/16 (25.0)
Remission	8/12 (66.7)	16/27 (59.3)	12/16 (75.0)
Immunosuppressive treatment before sampling:			
No treatment	3/9 (33.3)	17/24 (70.8)	5/9 (55.6)
Any treatment	6/9 (66.7)	7/24 (29.2)	4/9 (44.4)
Disease course:			
Monophasic	4/12 (33.3)	9/28 (32.1)	8/15 (53.3)
Relapsing	8/12 (66.7)	19/28 (67.9)	7/15 (46.7)
Clinical symptoms:			
Cerebral^a^	5/11 (45.5)	2/11 (18.2)	3/10 (30.0)
Opticospinal^b^	6/11 (54.5)	9/11 (81.8)	7/10 (70.0)

K-means cluster analysis was performed, comparing cluster 1 or 2 with cluster 3 as a reference. Significant findings are either labeled as * if p<0.05 or **p<0.01. MOGAD: myelin oligodendrocyte glycoprotein associated disease; MS: multiple sclerosis; NMOSD: neuromyelitis optica spectrum disorder.

^a^Cerebral symptoms included acute disseminated encephalomyelitis (ADEM)-like symptoms (ADEM, ADEM-optic neuritis, multiphasic disseminated encephalomyelitis or encephalitis). ^b^Opticospinal symptoms included optic neuritis and transverse myelitis.

Among those patients with clinical information available (min-max: 9-12 in cluster I, 11-28 in cluster II, 9-16 in cluster III), there was a tendency for more patients receiving treatment before sampling in cluster I (66.7% in cluster I vs 29.2% in cluster II vs 44.4% in cluster III). Opticospinal symptoms (optic neuritis, transverse myelitis) were the most dominant in all clusters.

**Figure 4:**
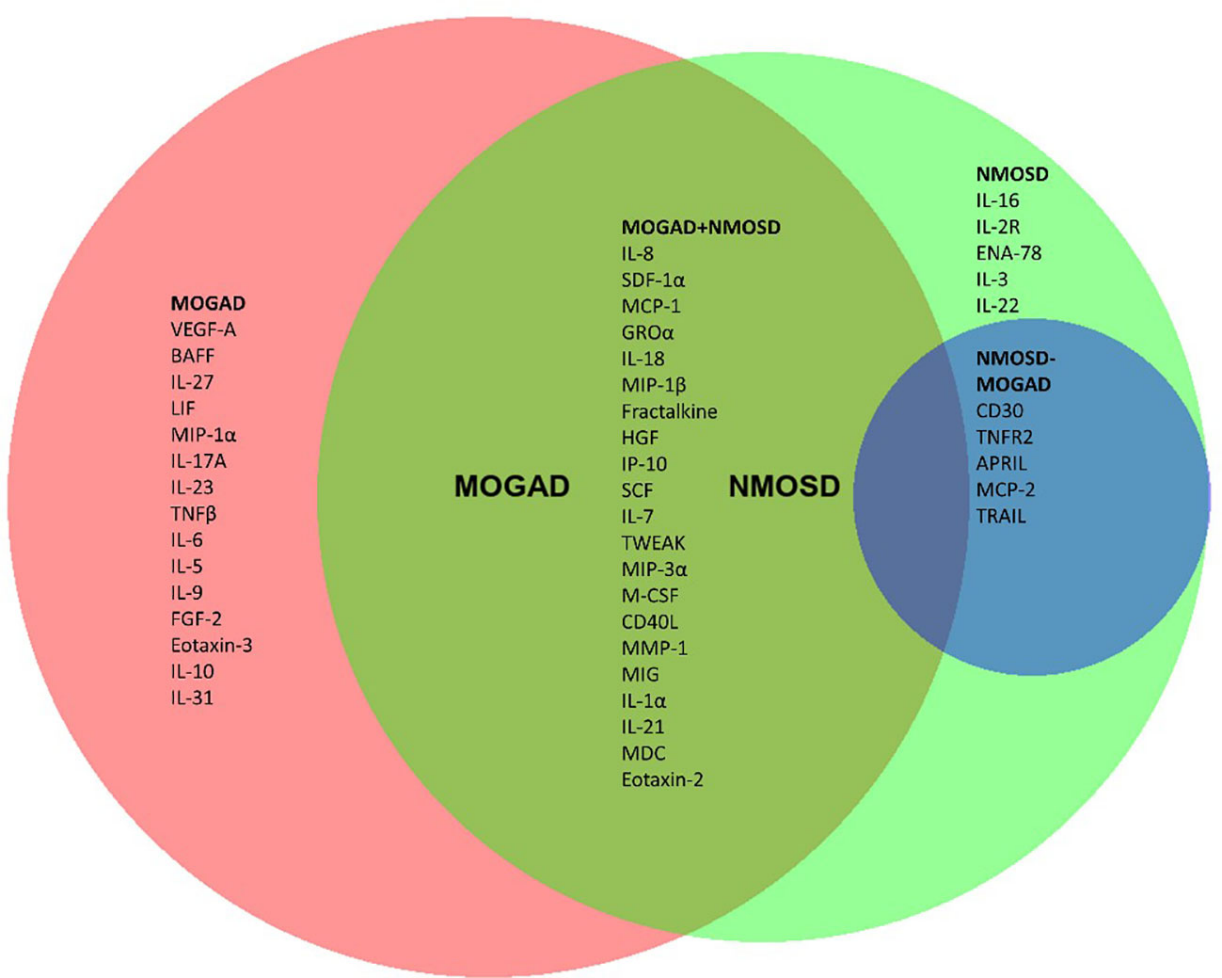
Cytokines, chemokines and related molecules significantly associated with MOGAD and/or NMOSD. **(A)** Venn diagram of significantly altered cytokines/chemokines/related molecules of pairwise Mann-Whitney U test comparison with MS patients. In red are those analytes which specifically differed in MOGAD patients compared to MS. In the green circle are those that were significantly altered in NMOSD patients compared to MS. In the overlapping part labeled as MOGAD+NMOSD are those that significantly differed for both MOGAD and NMOSD compared to MS. The blue circle shows those cytokines/chemokines that significantly differed in the direct comparison of NMOSD with MOGAD. APRIL, a proliferation-inducing ligand; BAFF, B cell activation factor; BLC, B lymphocyte chemoattractant; CD40L, CD40 ligand; ENA-78, epithelial neutrophil-activating peptide-78; FGF, fibroblast growth factor; G-CSF, granulocyte colony-stimulating factor; GM-CSF, granulocyte-macrophage colony-stimulating factor; GRO, growth-regulated oncogene; HGF, hepatocyte growth factor; IFN, interferon; IL, interleukin; IP, interferon-ɣ-induced protein; I-TAC, interferon-inducible T cell α-chemoattractant; LIF, leukemia inhibitory factor; MCP, monocyte chemoattractant protein; M-CSF, macrophage colony-stimulating factor; MDC, macrophage-derived chemokine; MIF, macrophage migration inhibitory factor; MIG, monokine induced by interferon-ɣ; MIP, macrophage inflammatory protein; MMP, matrix metalloproteinase; MOGAD, myelin oligodendrocyte glycoprotein associated disease; MS, multiple sclerosis; NGF, nerve growth factor; NMOSD, neuromyelitis optica spectrum disorder; SCF, stem cell factor; SDF, stromal cell-derived factor; TNF, tumor necrosis factor; TRAIL, TNF-related apoptosis-inducing ligand; TSLP, thymic stromal lymphopoietin; TWEAK, tumor necrosis factor-like weak inducer of apoptosis; VEGF, vascular endothelial growth factor.

Based on cluster analysis data, cluster I was specifically associated with increased levels of 11 IL-6/IL-17A associated cytokines/chemokines (FGF-2, IL-10, IL-17A, IL-23, IL-27, IL-31, IL-5, IL-6, IL-9, LIF, TNF-β). Patients associated with cluster II were found to have specifically higher levels of 31 cytokines/chemokines (APRIL, CD30, CD40L, ENA-78, Eotaxin-2, Fractalkine, GRO-α, HGF, IL-16, IL-18, IL-1α, IL-21, IL-22, IL-2R, IL-3, IL-7, IL-8, IP-10, MCP-1, MCP-2, M-CSF, MDC, MIG, MIP-1β, MIP-3α, MMP-1, SCF, SDF-1α, TNFR2, TRAIL, TWEAK), as shown in **Figure 4**. Cluster III was used as the reference cluster, as mainly MS patients were included.

### 3.3 Stability over time

The median follow-up time was 6 months (range 4.0-24.0) in NMOSD patients and in the MOGAD cohort 12 months (range 6.0-38.0) (**Table 1**). In NMOSD patients, all measured cytokines, chemokines and related molecules remained stable between baseline and follow-up time-point. In those patients with clinical information available, no influence of different treatments was found on the stability. In MOGAD patients, only three analytes significantly decreased over time (HGF, IL-7 and VEGF-A; **Supplementary Figure 3**).

### 3.4 Validation of selected cytokines, chemokines, and related molecules

#### 3.4.1 Validation of serum IL-6 levels

**Figure 5:**
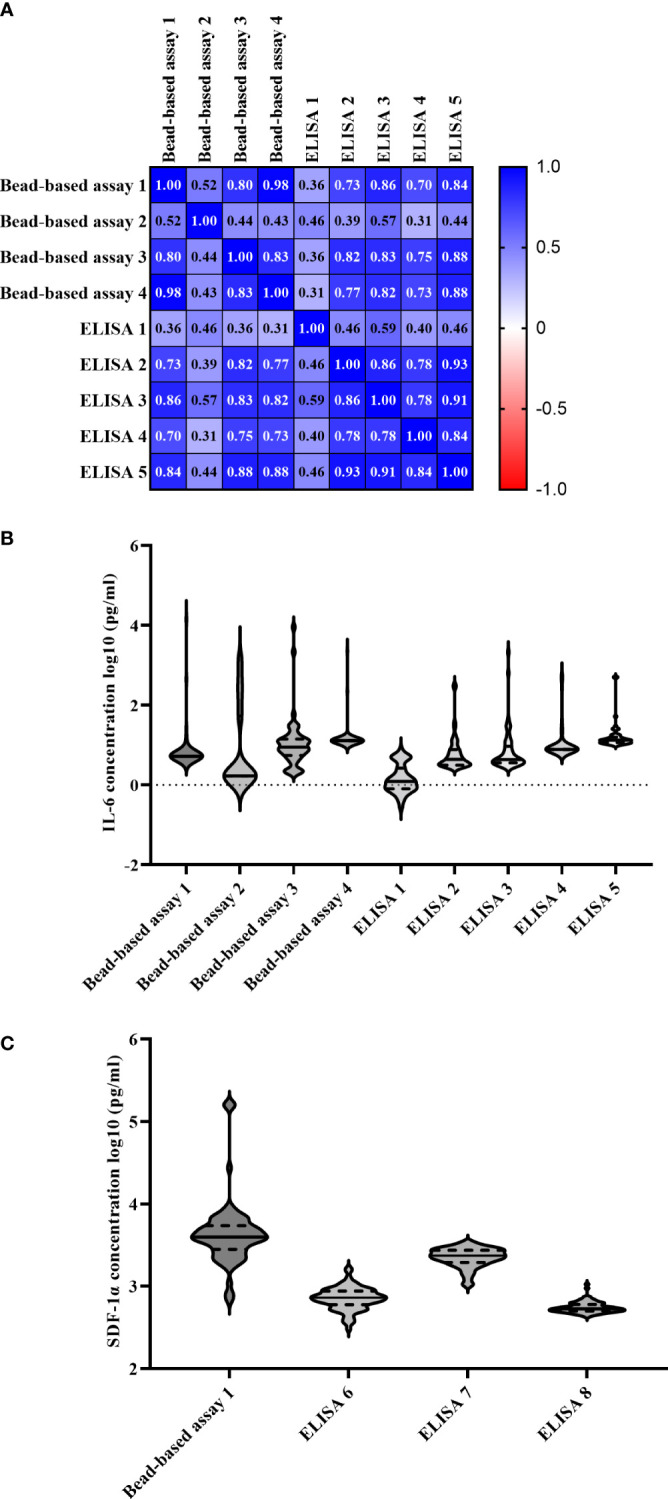
Comparability of multiple bead-based assays and ELISAs measuring IL-6 and SDF-1α levels. **(A)** Pearson correlation matrix of different IL-6 measuring bead-based assays using 50 randomly selected samples. **(B)** IL-6 levels (log10-transformed, pg/ml) of individual samples measured on all assays. The black horizontal lines represent the median concentrations with quartiles labeled as dashed lines. **(C)** SDF-1α levels (log10-transformed, pg/ml) of individual samples measured on all assays. The black horizontal lines represent the median concentrations with quartiles labeled as dashed lines. IL, interleukin; MOGAD, myelin oligodendrocyte glycoprotein associated disease; MS, multiple sclerosis; NMOSD, neuromyelitis optica spectrum disorder; SDF, stromal cell-derived factor.

Validation of IL-6 levels in 50 randomly selected samples revealed moderate to strong correlation coefficients ranging from 0.52-0.98 (median of 0.80) when comparing bead-based assay 1 with other bead-based assays and weak to strong correlation coefficients from 0.36-0.86 (median of 0.73) when comparing to ELISA-based assays (**Figure 5A**). Individual IL-6 concentrations of samples could not be reproduced on different assays as shown in **Figure 5B**.

#### 3.4.2 Validation of other analytes

Moreover, levels of 19 other cytokines, chemokines and related molecules (BLC, G-CSF, IFN-γ, IL-1β, IL-2, IL-4, IL-8, IL-10, IL-12p70, IL-13, IL-16, IL-17A, IL-18, IL-23, IP-10, MCP-1, MDC, SDF-1α, TNF-α) were validated with up to three different bead-based assays and three SDF-1α-measuring ELISA. Correlation for these cytokines and chemokines (except SDF-1α) ranged from the lowest correlation with -0.01 for IFN-γ up to the highest with 0.80 for IL-8 (**Supplementary Table 2**). The panel of cytokines, chemokines and related molecules that were measured with bead-based assays 2, 3 and 4 only partly overlapped (**Supplementary Table 3**). For validation of IL-16 levels, samples of 13 MS patients and 10 NMOSD patients were measured on bead-based assay 4 and no significant difference was found (p=0.136) between these two diseases.

For SDF-1α levels, results of bead-based assay 1 correlated weakly with ELISA 6-8 ranging from 0.04 with ELISA 8 up to 0.14 with ELISA 7 (**Supplementary Table 4**). Also, correlations within different ELISA manufacturers were ranging from a low degree of correlation between ELISA 7 and 8 up to a high degree of correlation between ELISA 6 and 8. Again, individual SDF-1α levels differed between assays (**Figure 5C**).

### 3.5 Altered cytokines, chemokines and related molecules in different diseases affecting the CNS

For better comparison of cytokines and chemokines associated with NMOSD and MOGAD with other neuroinflammatory disorders of the CNS we reviewed several studies using PubMed database on CSF and serum cytokines and chemokines and described patterns for autoantibody associated encephalitis, viral encephalitis, bacterial meningitis and neuropsychiatric systemic lupus erythematosus (NPSLE) in **Supplementary Table 5**. For ease of presentation, we have grouped cytokines and chemokines according to the typical signature of effector cells (Th1, Th2, T reg, Th17, B cell and other broad-spectrum cytokines/chemokines).

## 4 Discussion

In this study, we analyzed a panel of 65 cytokines, chemokines and related molecules using a bead-based multiplex assay (bead-based assay 1) in serum samples of patients with MOGAD or AQP4+ NMOSD and compared results with samples obtained from MS patients.

We found increased serum levels of the Th17-related cytokines IL-8, IL-21, IL-22, the inflammatory cytokines GRO-α MIP-1β, IP10 and APRIL in patients with AQP4+ NMOSD compared to MS. Similarly, in MOGAD a predominant involvement of Th17-related cytokines like IL-6, IL-8, IL-17A and IL-21 and high levels of broad-spectrum cytokines namely GRO-α and MIP-1β were found within the 36 significant hits that were clearly distinctive from MS. There was no difference of cytokine and chemokine levels between adult and pediatric MOGAD cases, which is in agreement with a previous study (10). All mentioned cytokines and chemokines (IL-6, IL-8, IL-17A, IL-21, IL-22, GRO-α, MIP-1β, IP10, APRIL) already had been shown to distinguish patients with autoantibody-associated diseases like NMOSD or MOGAD from MS (9, 10, 21–25).

Besides a Th17 dominant immune response, we could not demonstrate a definitive Th2 associated response in MOGAD and AQP4+ NMOSD analyzing serum samples, because IL-2, IL-4 and IL-13 were not increased in our population compared to MS patients. This result is in line with a study performed by Hofer, Mariotto, and colleagues (9), but partially discordant from data reported by Kaneko and colleagues, who observed CSF IL-13 significant different levels in comparison to MS (10).

As IL-6 targeting therapy satralizumab is approved for AQP4+ NMOSD (15, 16), IL-6 levels are of particular interest. We detected significantly increased serum levels of IL-6 in MOGAD patients and in a few patients with AQP4+ NMOSD. This might seem unexpected; however, high IL-6 levels in NMOSD were almost exclusively measured in CSF in former studies (9, 26) and those studies measuring serum IL-6 levels could not provide sufficient data to discriminate NMOSD from MS (9, 27). Furthermore, although a study by Haramati and colleagues could detect increased serum IL-6 levels during relapse compared to remission phase in NMOSD patients, we could not observe this difference in our study cohort for any analyte (28). One explanation might be the difference of included patients as Haramati and colleagues included also anti-AQP4 negative NMOSD patients with a much longer disease duration period and differences in treatment status may influence our findings. In another study, also higher IL-6 levels in NMOSD patients were found in those with greater EDSS (29), unfortunately, this clinical disability score was not retrospectively accessible in our cohort.

We observed an upregulation of 11 IL-6 and IL-17A related cytokines and chemokines in patients classified into cluster 1 using k-means cluster analysis. The majority of patients within this cluster were MOGAD patients with 70.6%, of whom 52.9% were children. Four out of ten MOGAD patients within cluster 1 with clinical data available had an ADEM-like presentation. We hypothesize that a preceding infectious prodrome, which is frequently seen before ADEM onset in children with MOGAD (30), may contribute to this coordinated upregulation of inflammatory cytokines, chemokines and related molecules, but may not adequately explain these findings as nearly all cytokine and chemokine levels remained stable in follow-up samples and preceding infections are thought to only be transient.

Studies analyzing cytokines and chemokines in different neurological diseases are very heterogenous in terms of cytokines and chemokines findings, control groups, cytokines/chemokines analysis and methodology as well as measured sample material (serum, plasma, CSF) and timing of sampling, which poses a challenge in comparing different studies. In other diseases the broad cytokines/chemokines analysis that we herein described have not been performed, thus limiting comparisons with other CNS diseases. Many of the cytokines associated with cluster 1 (IL-6, IL-5, IL-27, IL-23, IL-10, IL-17A) have been described in viral encephalitis or bacterial meningitis (31–43).

Both antibody-associated demyelinating diseases, AQP4+ NMOSD and MOGAD, showed a similar pattern of increased cytokines and chemokines compared to MS. Levels of five analytes were significantly higher in NMOSD patients, namely B cell-related cytokine APRIL, apoptosis inducing cytokine TRAIL, factor TNFR2, leukocyte recruiting MCP-2 and CD30 a member of the tumor necrosis factor receptor superfamily. Others have found similar cytokine levels comparing MOGAD with AQP4+ NMOSD, but most of these results are limited by the lower number of cytokine analyzed (10) or the absence of a clear comparison between autoantibody-associated diseases (9). One study did not find a difference of MCP-2 serum concentrations between AQP4+ NMOSD and MOGAD patients using ELISA and including less patients, but showed a significant difference for CXCL6 (GCP-2), which we did not measure (44).

In order to use cytokines and chemokines as disease-specific markers for instance for therapeutic targets, stability over time needs to be evaluated. Our study assessed this issue and all cytokines, chemokines and related molecules remained stable for up to 12 months except for HGF, IL-7 and VEGF-A levels in MOGAD patients, which significantly declined. One explanation for this might be, that the stability between different cytokines and chemokines differs (e.g. IL-2, IL-4, IL-12 and IL-18 are classified as relatively stable at -80°C (45)). Therefore, as this study was conducted as a multicenter study, confounding factors like effects of storage, differences in collection tubes or timing before processing, may have negatively influenced the stability of these three analytes in some samples of our MOGAD cohort.

In this study, we have demonstrated a strong correlation of serum IL-6 levels using four bead-based assays and five ELISA. Unfortunately and a major limitation of this study, we have not used an international IL-6 standard on all assays to validate comparability and our validation part only covered a few aspects of a full validation process. However, it must be noted that absolute concentrations of serum IL-6 differed between assays in our study. This might be explained by the fact that antibodies from different manufacturers might have different epitope-binding sites and the influence from plasma proteins is not necessarily the same between different assays. Furthermore, different instruments/platforms and reagents might influence results. Consequently, the levels of cytokine and chemokine concentrations cannot be compared directly between different assays also as they had different dynamic ranges. A study by Khan et al. measuring five cytokines in serum of healthy individuals after i.v. injection with endotoxin found differences of absolute levels between their assays. Unfortunately, they have not used the same 65-plex kit as we did, but as they used bead-based multiplex kits from LINCO Research, Bio-Rad Laboratories and R&D Systems and compared results with ELISA, we do think that these results are comparable to ours (46). Another study reported that plasma proteins, heterophilic- and autoantibodies might cause false positive and false negative signals by binding to assay antibodies used for capturing or detection or by binding directly to the antigen (47), which may contribute to the lack of comparability of absolute cytokine concentrations using different assays. For other blood-based markers like neurofilament light chain, also limited comparability between assays was published showing strong correlations but differing absolute concentrations (48). Our findings might explain the above-mentioned previous discrepant findings of cytokine and chemokine levels in the literature as multiple methods to measure cytokines and chemokines are available and comparability of absolute concentrations between different assays as we showed, is limited.

Our study has several strengths. To our knowledge, it is the first study analyzing such a broad spectrum of different cytokines and chemokines (n=65) in serum of patients with a diagnosis of either AQP4+ NMOSD, MOGAD or MS. In contrast to many other studies, we measured follow-up samples after a median of 6-12 months to assess the stability of these cytokines and chemokines. The use of serum samples instead of CSF has the major advantage that serum is less invasive to obtain and allows several measurements over time. Moreover, unique at our study is that we also assessed comparability of measured cytokine and chemokine levels between different manufacturers and between different analytic techniques comparing bead-based assays with several different ELISA.

The limitations of this study include the retrospective design, which partially limited the availability of clinical information and may impact our conclusions in relation to clinical data. As a consequence, the influence of different immunomodulatory/immunosuppressive treatments or corticosteroid infusions on cytokine and chemokine levels could not be assessed properly because no information on treatment history was available in approximately half of AQP4+ NMOSD and MOGAD patients and no information on previous infections/vaccinations was available in any of them. Especially, in our NMOSD and MOGAD cohort many participants from whom we had this information available, received treatment, whereas in MS patients only five patients were treated, which may be a potential confounding factor. Although it has already been shown that some treatments are influencing cytokine/chemokine levels (49, 50), a further stratification of different treatments was not feasible in our cohort due to the limited number of cases per group as demonstrated in **Table 1**.

Next, as endogenous IL-6 expression follows a stringent circadian rhythm (51) and therefore blood collections for IL-6 measurements at distance time points need to be synchronized, our retrospective study design did not allow this. In addition, availability of follow-up samples after approximately 6-12 months was limited hence; only 25 follow-up samples of NMOSD patients could be included. Furthermore, median time until follow-up samples differed between the two autoantibody-associated diseases. Consequently, early follow-up samples after 4 months in AQP4+ NMOSD or 6 months in MOGAD might have an impact on the results of stability. However, nearly all analytes remained stable, also in those patients with long follow-up periods.

In conclusion, our study gives a detailed overview of cytokine and chemokine profiles in the autoantibody-associated diseases MOGAD and AQP4+ NMOSD compared to MS. Furthermore, our data showed high correlation for IL-6 between multiple bead-based assays and ELISA. Because absolute IL-6 levels in our study differed between different assays, we recommend being cautious when calculating cut-off values for individual cytokines or chemokines to distinguish different diseases or to assess treatment efficacy. Although presumably mainly due to the retrospective design of our study and incomplete clinical information of our study cohort, differences in cytokine and chemokine levels could not be found in the context of disease activity or clinical outcome; therefore, more extensive studies are needed to hopefully find a panel of cytokines and chemokines to monitor inflammation and response to treatment.

## Data availability statement

The original contributions presented in the study are included in the article/**Supplementary Material**. Further inquiries can be directed to the corresponding author.

## Ethics statement

The present study was approved by the ethical committees of the Medical University of Innsbruck, Austria (AM3041A and AM4059), Medical University of Vienna (EK 1636/2019 and 1123/2015) and 23 samples were obtained from the Neuropathology-Verona biobank. All patients or their legal representatives gave written informed consent to diagnostic procedures and biological sample storage for research purposes.

## Author contributions

AB and DR performed experiments, analyzed and interpreted data. AB prepared the main body of the manuscript. MR designed the study, supervised the work, interpreted data and participated in the preparation of the manuscript and prepared with AB Figures and Tables. KB, AD, CL, E-MW, HH, FD, TB, RH, KR, SM and MR provided patient material and all revisited the article critically for important intellectual content. All authors approved the final version of the manuscript.

## Funding

This study was supported by VASCage – Research Centre on Vascular Ageing and Stroke, Medical University Innsbruck and Roche Austria through a tripartite contract. VASCage is a COMET Centre within the Competence Centers for Excellent Technologies (COMET) programme and funded by the Federal Ministry for Climate Action, Environment, Energy, Mobility, Innovation and Technology the Federal Ministry of Labour and Economy, and the federal states of Tyrol, Salzburg and Vienna. COMET is managed by the Austrian Research Promotion Agency (Österreichische Forschungsförderungsgesellschaft).

## Acknowledgments

The authors want to thank Angus Byars, Paris Sidiropoulos, Sherman Jia, Shervin Gholizadeh, Ivana Vodopivec, Jillian Smith and Veronica Anania from Roche and Genentech for supporting this research and providing valuable expertise that greatly assisted this paper. We are also immensely grateful to Kathrin Schanda (Innsbruck, Austria) for performing cell-based assays analyzing antibodies targeting MOG and AQP4. Furthermore, we like to thank all patients and clinicians who included patients and took blood for research purposes. Therefore, we especially want to thank Markus Breu, Paul Rommer, Barbara Bajer-Kornek und Helmut Rauschka.

## Conflict of interest

AB is an employee of VASCage – Research Centre on Vascular Ageing and Stroke and has participated in meetings sponsored by or received travel funding from Novartis, Sanofi Genenzyme, Merck, Almirall and Biogen. KB has participated in meetings sponsored by and received travel funding from Roche, Biogen and Teva. HH has participated in meetings sponsored by, received speaker honoraria or travel funding from Bayer, Biogen, Celgene, Merck, Novartis, Sanofi-Genzyme, Siemens and Teva, and received honoraria for consulting Biogen, Celgene, Novartis and Teva. FD has participated in meetings sponsored by or received honoraria for acting as an advisor/speaker for Alexion, Almirall, Biogen, Celgene, Janssen, Merck, Novartis, Roche and Sanofi-Genzyme. His institution received scientific grants from Biogen and Sanofi-Genzyme.TB has participated in meetings sponsored by and received honoraria lectures, advisory boards, consultations from pharmaceutical companies marketing treatments for MS: Allergan, Bayer, Biogen, Bionorica, BMS/Celgene, GSK, GW/Jazz Pharma, Horizon, Janssen-Cilag, MedDay, Merck, Novartis, Octapharma, Roche, Sandoz, Sanofi-Genzyme, Teva and UCB. His institution has received financial support in the past 12 months by unrestricted research grants Biogen, Bayer, BMS/Celgene, Merck, Novartis, Roche, Sanofi-Genzyme, Teva and for participation in clinical trials in multiple sclerosis sponsored by Alexion, Bayer, Biogen, Merck, Novartis, Octapharma, Roche, Sanofi-Genzyme, Teva. RH has received honoraria for lectures from Novartis and Biogen. KR has served as consultant for the PARADIGM Study/Novartis without payment. SM received speaker honoraria from Novartis and Biogen. MR was supported by a research support from Euroimmun and Roche. The University Hospital and Medical University of Innsbruck Austria, employer of MR receives payments for antibody assays MOG, AQP4, and other autoantibodies and for MOG and AQP4 antibody validation experiments organized by Euroimmun Lübeck, Germany.

The remaining authors declare that the research was conducted in the absence of any commercial or financial relationships that could be construed as a potential conflict of interest.​

The authors declare that this study received funding from Roche Austria. The funder was involved in the study design and critical revision of the article for important intellectual content.

## Publisher’s note

All claims expressed in this article are solely those of the authors and do not necessarily represent those of their affiliated organizations, or those of the publisher, the editors and the reviewers. Any product that may be evaluated in this article, or claim that may be made by its manufacturer, is not guaranteed or endorsed by the publisher.
