# Supplementary material for: Increased peripheral inflammatory responses in myelin oligodendrocyte glycoprotein associated disease and aquaporin-4 antibody positive neuromyelitis optica spectrum disorder
Source: Front Immunol. 2022 Nov 14;13:1037812. doi: 10.3389/fimmu.2022.1037812 (PMC9703059; doi:10.3389/fimmu.2022.1037812)
Supplement: Supplementary file 1 [file DataSheet_1.pdf]

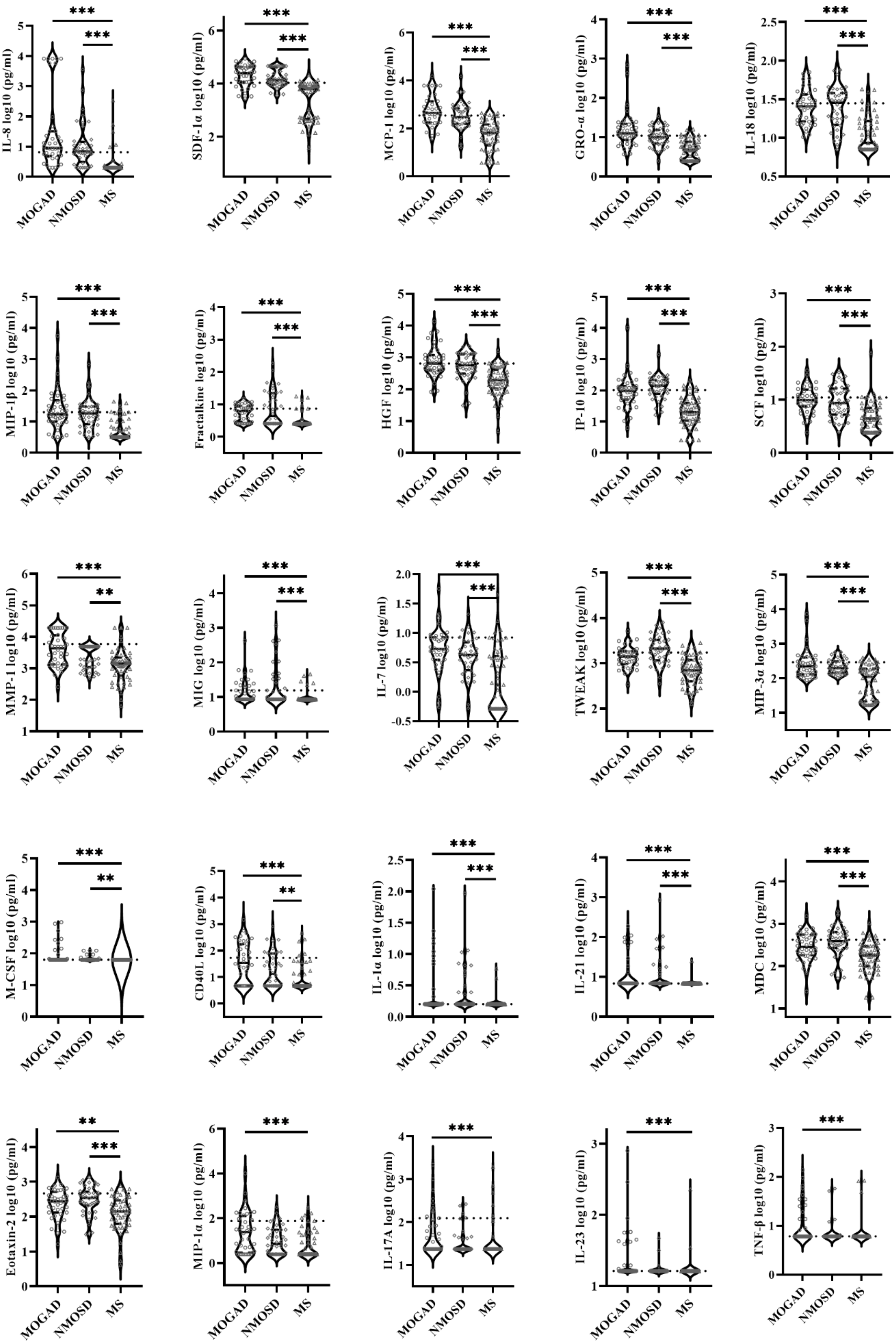
IL-8 log10 (pg/ml)
SDF-1α log10 (pg/ml)
MCP-1 log10 (pg/ml)
GRO-α log10 (pg/ml)
IL-18 log10 (pg/ml)
MIP-1β log10 (pg/ml)
Fractalkine log10 (pg/ml)
HGF log10 (pg/ml)
IP-10 log10 (pg/ml)
SCF log10 (pg/ml)
MMP-1 log10 (pg/ml)
MIG log10 (pg/ml)
IL-7 log10 (pg/ml)
TWEAK log10 (pg/ml)
MIP-3α log10 (pg/ml)
M-CSF log10 (pg/ml)
CD40L log10 (pg/ml)
IL-1α log10 (pg/ml)
IL-21 log10 (pg/ml)
MDC log10 (pg/ml)
Eotaxin-2 log10 (pg/ml)
MIP-1α log10 (pg/ml)
IL-17A log10 (pg/ml)
IL-23 log10 (pg/ml)
TNF-β log10 (pg/ml)
MOGAD
NMOSD
MS
***
**

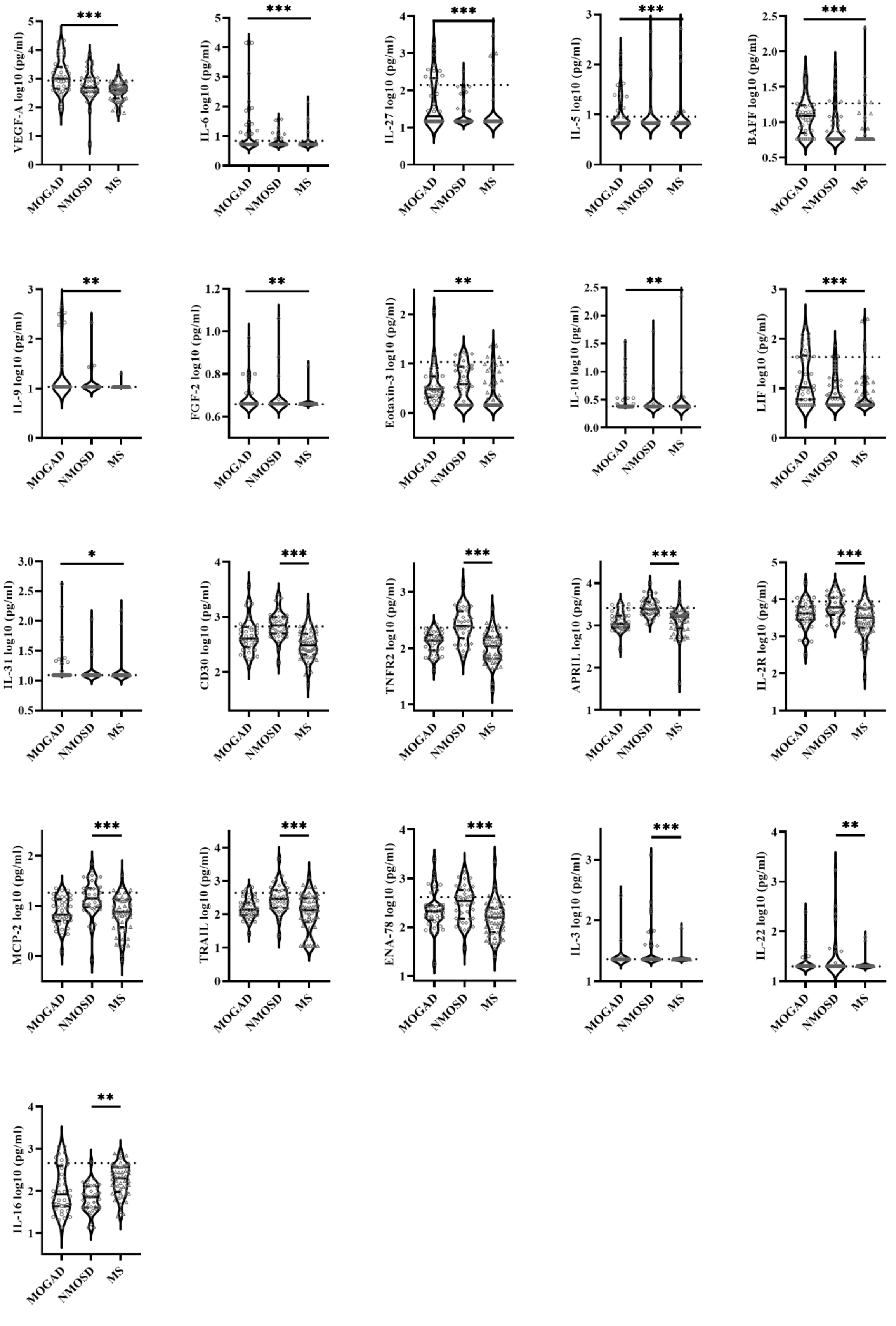

Supplementary Figure 1. Violin plots of significantly altered cytokines and chemokines compared to multiple sclerosis (MS) patients. Individual values for aquaporin-4 antibody positive neuromyelitis optica spectrum disorder (NMOSD) patients, myelin oligodendrocyte

glycoprotein-associated disease (MOGAD) patients and MS patients are shown as circles, medians are shown as thick horizontal line and quartiles as dashed line. The long horizontal dotted lines represent the cut-off values to discriminate NMOSD and MOGAD from MS patients with 90% specificity. Between group comparisons, comparing MOGAD or NMOSD with MS, were calculated using Mann Whitney U test with two-stage step-up (Benjamini, Krieger and Yekutieli) and a false discovery rate (FDR) of 1%. Significant differences (MOGAD and NMOSD versus MS) are shown in the graphs, *** indicates a p-value <0.001, ** p-value <0.01, * p-value <0.05.

APRIL: a proliferation-inducing ligand; BAFF: B cell activation factor; BLC: B lymphocyte chemoattractant; CD40L: CD40 ligand; ENA-78: epithelial neutrophil-activating peptide-78; FGF: fibroblast growth factor; G-CSF: granulocyte colony-stimulating factor; GM-CSF: granulocyte-macrophage colony-stimulating factor; GRO: growth-regulated oncogene; HGF: hepatocyte growth factor; IFN: interferon; IL: interleukin; IP: interferon-ɣ-induced protein; I-TAC: interferon-inducible T cell α-chemoattractant; LIF: leukemia inhibitory factor; MCP: monocyte chemoattractant protein; M-CSF: macrophage colony-stimulating factor; MDC: macrophage-derived chemokine; MIF: macrophage migration inhibitory factor; MIG: monokine induced by interferon-ɣ; MIP: macrophage inflammatory protein; MMP: matrix metalloproteinase; MOGAD: myelin oligodendrocyte glycoprotein associated disease; MS: multiple sclerosis; NGF: nerve growth factor; NMDAR: N-methyl-D-aspartate receptor; NMOSD: neuromyelitis optica spectrum disorder; SCF: stem cell factor; SDF: stromal cell-derived factor; TNF: tumor necrosis factor; TRAIL: TNF-related apoptosis-inducing ligand; TSLP: thymic stromal lymphopoietin; TWEAK: tumor necrosis factor-like weak inducer of apoptosis; VEGF: vascular endothelial growth factor

.

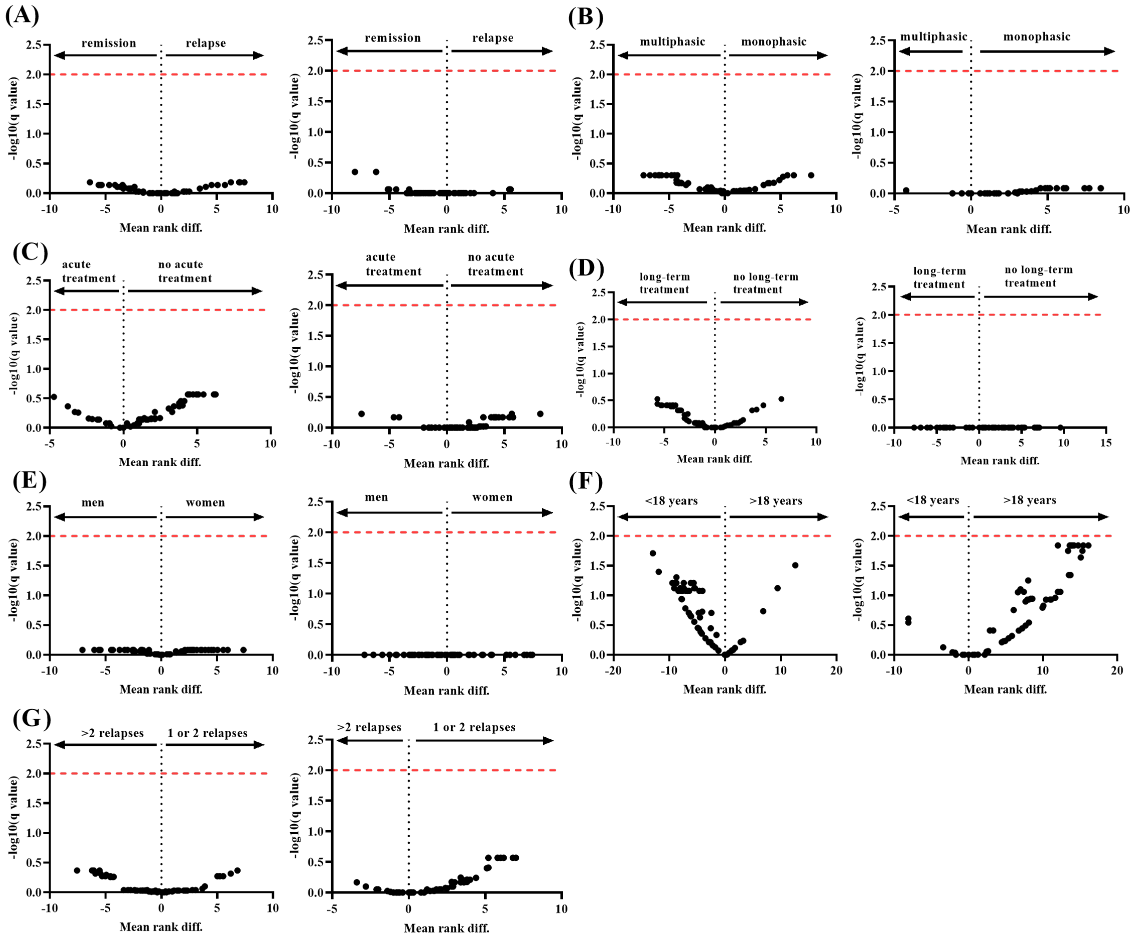

Supplementary Figure 2. Clinical differences within MOGAD and NMOSD patients. Volcano Plot of Mann-Whitney tests after two-stage step-up (Benjamini, Krieger and Yekutieli) and FDR of 1% correction for multiple comparisons showing levels of 65 cytokines, chemokines and related molecules in MOGAD (left) and NMOSD (right) patients **A** during remission or relapse. **B** comparing relapsing vs monophasic disease courses. **C** receiving acute treatment or not before sample was taken. **D** receiving long-term treatment or not. **E** comparing women with men. **F** comparing children aged younger 18 years with adults. **G** having a maximum of two relapses compared with those having at least three relapses. The red dotted line indicates the significance level of $p<0.05$.

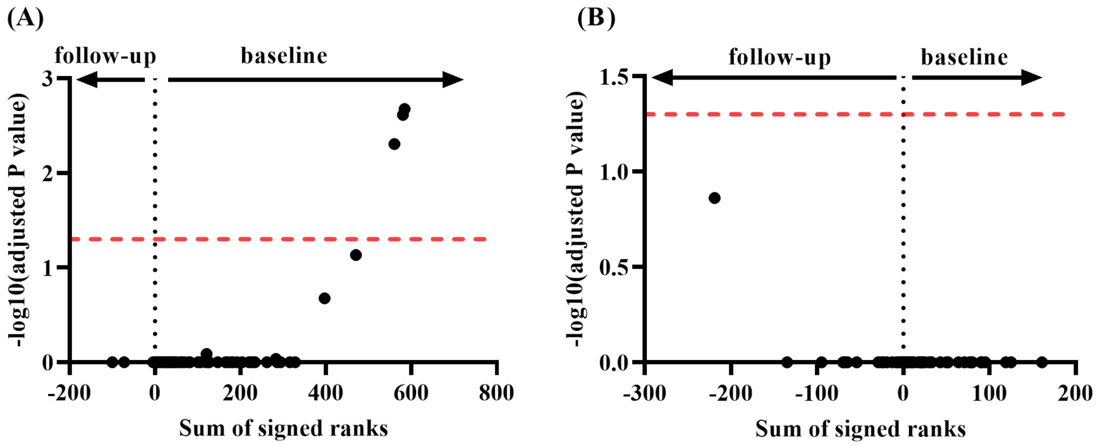

Supplementary Figure 3. Stability over time. Volcano plots of Wilcoxon matched-pairs signed rank test after Bonferroni-Dunn correction comparing serum levels of 65 cytokines, chemokines and related molecules between baseline and follow-up samples in **A** MOGAD and **B** NMOSD patients. The red dotted line indicates the significance level of p<0.05.

Supplementary Table 1. Dysregulated cytokine/chemokine concentrations, which distinguished NMOSD and MOGAD from MS with 90% specificity.

Cytokine/ chemokine	90th percentile of MS patients (pg/ml)	NMOSD	MOGAD	MS	p-value (NMOSD vs MS)	p-value (MOGAD vs MS)
APRIL	2567.92	18/40(45%)	5/40 (13%)	5/54 (9%)	<0.001	
BAFF	18.34	6/40 (15%)	6/40 (15%)	5/54 (9%)		<0.001
CD30	671.02	21/40 (53%)	9/40 (23%)	5/54 (9%)	<0.001	
CD40L	53.21	11/40 (28%)	17/40 (43%)	5/54 (9%)	0.002	<0.001
ENA-78	411.74	17/40 (43%)	8/40 (20%)	5/54 (9%)	<0.001	
Eotaxin-2	466.51	13/40 (33%)	10/40 (25%)	5/54 (9%)	<0.001	0.007
Eotaxin-3	10.86	6/40 (15%)	3/40 (8%)	5/54 (9%)		0.004
FGF-2	4.54	4/40 (10%)	9/40 (23%)	1/54 (2%)		0.001
Fractalkine	7.41	18/40 (45%)	16/40 (40%)	4/54 (7%)	<0.001	<0.001
GRO-α	10.88	16/40 (40%)	28/40 (70%)	5/54 (9%)	<0.001	<0.001
HGF	647.67	19/40 (48%)	20/40 (50%)	5/54 (9%)	<0.001	<0.001
IL-10	2.40	6/40 (15%)	12/40 (30%)	4/54 (7%)		0.006
IL-16	455.41	1/40 (3%)	8/40 (20%)	5/54 (9%)	<0.001	
IL-17A	123.56	4/40 (10%)	9/40 (23%)	5/54 (9%)		<0.001
IL-18	28.04	20/40 (50%)	16/40 (40%)	5/54 (9%)	<0.001	<0.001
IL-1α	1.59	13/40 (33%)	11/40 (28%)	2/54 (4%)	<0.001	<0.001
IL-21	6.84	11/40 (28%)	9/40 (23%)	1/54 (2%)	<0.001	<0.001
IL-22	19.95	9/40 (23%)	6/40 (15%)	2/54 (4%)	0.006	
IL-23	16.19	3/40 (8%)	12/40 (30%)	2/54 (4%)		<0.001
IL-27	139.41	2/40 (5%)	12/40 (30%)	5/54 (9%)		<0.001
IL-2R	8697.27	14/40 (35%)	6/40 (15%)	5/54 (9%)	<0.001	
IL-3	23.12	10/40 (25%)	3/40 (8%)	1/54 (2%)	<0.001	
IL-31	12.33	3/40 (8%)	10/40 (25%)	3 (54 (6%)		0.010
IL-5	9.09	5/40 (13%)	13/40 (33%)	5/54 (9%)		<0.001
IL-6	6.92	8/40 (20%)	15/40 (38%)	5/54 (9%)		<0.001
IL-7	8.39	6/40 (15%)	10/40 (25%)	4/54 (7%)	<0.001	<0.001
IL-8	6.51	21/40 (53%)	25/40 (63%)	5/54 (9%)	<0.001	<0.001
IL-9	10.67	3/40 (8%)	8/40 (20%)	1/54 (2%)		0.001
IP-10	101.99	26/40 (65%)	19/40 (48%)	5/54 (9%)	<0.001	<0.001
LIF	42.83	3/40 (8%)	11/40 (28%)	5/54 (9%)		<0.001
MCP-1	346.64	18/40 (45%)	26/40 (65%)	5/54 (9%)	<0.001	<0.001
MCP-2	18.37	14/40 (35%)	3/40 (8%)	5/54 (9%)	<0.001	
M-CSF	63.38	7/40 (18%)	11/40 (28%)	0/54 (0%)	0.002	<0.001
MDC	420.40	18/40 (45%)	14/40 (35%)	5/54 (9%)	<0.001	<0.001
MIG	15.56	14/40 (35%)	11/40 (28%)	5/54 (9%)	<0.001	<0.001
MIP-1α	76.10	4/40 (10%)	12/40 (30%)	5/54 (9%)		<0.001
MIP-1β	20.14	19/40 (48%)	19/40 (48%)	5/54 (9%)	<0.001	<0.001
MIP-3α	291.42	11/40 (28%)	13/40 (33%)	5/54 (9%)	<0.001	<0.001
MMP-1	5898.92	0/40 (0%)	14/40 (35%)	5/54 (9%)	0.005	<0.001
SCF	10.99	16/40 (40%)	17/40 (43%)	5/54 (9%)	<0.001	<0.001
SDF-1α	10814.51	26/40 (65%)	32/40 (80%)	5/54 (9%)	<0.001	<0.001
TNF-β	6.08	5/40 (13%)	14/40 (35%)	3 (54 (6%)		<0.001
TNFR2	230.93	22/40 (55%)	2/40 (5%)	5/54 (9%)	<0.001	

TRAIL	437.95	14/40 (35%)	3/40 (8%)	5/54 (9%)	<0.001	
TWEAK	1731.21	24/40 (60%)	12/40 (30%)	5/54 (9%)	<0.001	<0.001
VEGF-A	859.19	12/40 (30%)	22/40 (55%)	5/54 (9%)		<0.001

APRIL: a proliferation-inducing ligand; BAFF: B cell activation factor; BLC: B lymphocyte chemoattractant; CD40L: CD40 ligand; ENA-78: epithelial neutrophil-activating peptide-78; FGF: fibroblast growth factor; G-CSF: granulocyte colony-stimulating factor; GM-CSF: granulocyte-macrophage colony-stimulating factor; GRO: growth-regulated oncogene; HGF: hepatocyte growth factor; IFN: interferon; IL: interleukin; IP: interferon-ɣ-induced protein; I-TAC: interferon-inducible T cell α-chemoattractant; LIF: leukemia inhibitory factor; MCP: monocyte chemoattractant protein; M-CSF: macrophage colony-stimulating factor; MDC: macrophage-derived chemokine; MIF: macrophage migration inhibitory factor; MIG: monokine induced by interferon-ɣ; MIP: macrophage inflammatory protein; MMP: matrix metalloproteinase; MOGAD: myelin oligodendrocyte glycoprotein associated disease; MS: multiple sclerosis; NGF: nerve growth factor; NMDAR: N-methyl-D-aspartate receptor; NMOSD: neuromyelitis optica spectrum disorder; SCF: stem cell factor; SDF: stromal cell-derived factor; TNF: tumor necrosis factor; TRAIL: TNF-related apoptosis-inducing ligand; TSLP: thymic stromal lymphopoietin; TWEAK: tumor necrosis factor-like weak inducer of apoptosis; VEGF: vascular endothelial growth factor

Supplementary Table 2. Pearson's correlation coefficients comparing levels of cytokines, chemokines and related molecules measured with bead-based assay 1 with other bead-based assays.

Analyte	Bead-based assay 1	Bead-based assay 2	Bead-based assay 3	Bead-based assay 4
BLC	1.000	n.a.	n.a.	0.218
G-CSF	1.000	0.312	n.a.	n.a.
IFN-γ	1.000	-0.012	0.455	n.a.
IL-10	1.000	0.224	0.424	n.a.
IL-12p70	*	*	*	*
IL-13	*	*	n.a.	n.a.
IL-16	1.000	n.a.	n.a.	0.214
IL-17A	1.000	0.100	0.104	*
IL-18	1.000	n.a.	-0.028	n.a.
IL-1β	1.000	0.315	0.799	n.a.
IL-12	1.000	0.217	n.a.	n.a.
IL-23	1.000	n.a.	0.261	n.a.
IL-4	1.000	0.091	n.a.	n.a.
IL-8	1.000	n.a.	0.462	0.802
IP-10	1.000	n.a.	n.a.	0.077
MCP-1	1.000	0.357	0.325	n.a.
MDC	1.000	n.a.	n.a.	0.684
TNF-α	1.000	0.103	0.564	n.a.

BLC: B lymphocyte chemoattractant; G-CSF: granulocyte colony-stimulating factor; IFN: interferon; IL: interleukin; IP: interferon-ɣ-induced protein; MCP: monocyte chemoattractant protein; MDC: macrophage-derived chemokine; n.a.: not analyzed; TNF-related apoptosis-inducing ligand;* analyte not detectable

Supplementary Table 3. Pearson's correlation coefficients comparing bead-based assays 2, 3 and 4.

Reference	Analyte	Bead-based assay 1	Bead-based assay 2	Bead-based assay 3	Bead-based assay 4
Bead-based assay 2	G-CSF	0.312	1.000	n.a.	n.a.
Bead-based assay 2	IFN-γ	-0.012	1.000	0.120	n.a.
Bead-based assay 2	IL-10	0.224	1.000	0.264	n.a.
Bead-based assay 2	IL-12p70	*	1.000	0.040	n.a.
Bead-based assay 2	IL-13	*	1.000	n.a.	n.a.
Bead-based assay 2	IL-17A	0.100	1.000	-0.112	*
Bead-based assay 2	IL-1β	0.315	1.000	0.285	n.a.
Bead-based assay 2	IL-12	0.217	1.000	n.a.	n.a.
Bead-based assay 2	IL-4	0.091	1.000	n.a.	n.a.
Bead-based assay 2	MCP-1	0.357	1.000	0.569	n.a.
Bead-based assay 2	TNF-α	0.103	1.000	0.205	n.a.
Bead-based assay 3	IL-18	-0.028	n.a.	1.000	n.a.
Bead-based assay 3	IL-23	0.261	n.a.	1.000	n.a.
Bead-based assay 3	IL-8	0.462	n.a.	1.000	0.695
Bead-based assay 4	BLC	0.218	n.a.	n.a.	1.000
Bead-based assay 4	IL-16	0.214	n.a.	n.a.	1.000
Bead-based assay 4	IP-10	0.077	n.a.	n.a.	1.000
Bead-based assay 4	MDC	0.684	n.a.	n.a.	1.000

BLC: B lymphocyte chemoattractant; G-CSF: granulocyte colony-stimulating factor; IFN: interferon; IL: interleukin; IP: interferon-ɣ-induced protein; MCP: monocyte chemoattractant protein; MDC: macrophage-derived chemokine; n.a.: not analyzed; TNF: tumor necrosis factor; * analyte not detectable

Supplementary Table 4. Pearson's correlation coefficients comparing SDF-1α levels.

	Bead-based assay 1	ELISA 6	ELISA 7	ELISA 8
Bead-based assay 1	1.000	0.095	0.137	0.043
ELISA 6	0.095	1.000	0.397	0.693
ELISA 7	0.137	0.397	1.000	0.049
ELISA 8	0.043	0.693	0.049	1.000

SDF: stromal cell-derived factor

Supplementary Table 5. Cytokine-chemokine profile in different diseases affecting the central nervous system, either measured in blood (serum or plasma) (pink) or CSF (blue). We listed only cytokines and chemokines that are significantly different compared to control groups, which are the following: **A** healthy controls without infections/meningitis, **B** MS and NMOSD controls, **C** MS, **D** other non-inflammatory neurological disorders or non-SLE control group, **E** meningoencephalic phase versus 2 months follow-up of these patients, **F** meningoencephalic phase versus early phase without CNS symptoms, **G** non-NPSLE patients.

Immune effector cells	Cytokines/ chemokines	MOGAD	NMOSD	Anti-NMDAR encephalitis	Viral encephalitis	Bacterial meningitis	NPSLE
Th1	IL-12p70					A (1); A (1)	B (2)
	TNF-α	C, D (3)		D (4, 5)	E (6); D (4, 7)	D (7), A (1)	B (2)
	MIG	C	C		E (6), F (8); D (4)		
	IP-10	C; D (3)	C; B (8)	D (4, 5)	F (8); D (4), A (9)		
	IFN-γ	C, D (3)	C (3)	D (4, 5)	F (8), A (10); D (7)	D (7), A (11)	B (2)
	I-TAC				F (8)		
Th2	IL-2			D (12)		D (7)	B (2)
	IL-4						
	IL-5	C			A (13)		B (2)
	IL-13	C, D (3)	C, D (3)	D (4)	D (4, 14)	D (15)	B (2)
T reg	IL-10	C; C (3)	C (3)	D (4, 5)	A (10); D (4, 7)	A (16, 7); D (7, 10), A (1, 11)	G (17), B (2)
Th17	IL-6	C; C, D (3)	C, D (3)	C (18); D (4)	A (13, 10); D (4, 14, 7)	D (7), A (1); D (7, 10), A (1, 11)	D (19); G (17)
	IL-8	C; C, D (3)	C; C, D (3)	C (18)	A (13); D (4, 14)	A (1)	G (17)
	IL-17A	C				A (16)	B (2)
	IL-23	C		D (4)	D (4)	A (20)	
B cells	SDF-1α	C	C	D (4)	D (4)		
	BLC			D (4, 5)	F (8); D (4)		
	APRIL	C (18)	C	C (18)			
	BAFF	C			F (8); D (4)		

Broad spectrum/ Others	IFN-α	C				D (4)	E (6), F (8)	D (4), A (9)			
	MCP-1	C	C (3)	C	C (3)		A (13), E (6), F (8)			G (21, 17)	
	MIP-1β	C	D (3)	C	D (3)	C (18)					
	IL-1α	C	C, D (3)	C	C, D (3)	D (4)	D (4)				
	GRO-α	C		C		C (18)	F (8)				
	Fractalkine	C		C						G (22)	
	IL-18	C		C			D (14)		A (20)		
	TNF-β	C		C							
	IL-21	C		C			E (6)				
	MDC	C		C		D (5)					
	IL-9	C	D (3)							B (2)	
	FGF-2	C	D (3)	D (3)						A (23)	B (2)
	Eotaxin-3	C									
	Eotaxin-2	C		C							
	Eotaxin									B (2)	
	IL-31	C									
	HGF	C		C			A (13)				
	SCF	C		C							
	VEGF-A	C					A (13), F (8)				
	IL-7	C		C			D (14)			B (2)	
	TWEAK	C		C							
	MIP-3α	C		C							
	M-CSF	C		C							
	G-CSF									G (17)	
	GM-CSF						A (13)			B (2)	
	CD40L	C		C							
	MMP-1	C		C							
	IL-27	C					E (6)				
	LIF	C									
	MIP-1α	C	C, D (3)	C, D (3)		D (5)			D (15)		

CD30	C					
TNFR2	C					
IL-16	C	D (5)				
IL-2R	C					
MCP-2	C	D (5)				
TRAIL	C					
ENA-78	C					
IL-3	C					
IL-22	C					
IL-15						B (2)
IL-20						
MIF						
TSLP						
MCP-3						
NGF-β						
IL-1β		D (4)	D (4, 14)	A (16)	A (1)	A (24)

APRIL: a proliferation-inducing ligand; BAFF: B cell activation factor; BLC: B lymphocyte chemoattractant; CD40L: CD40 ligand; ENA-78: epithelial neutrophil-activating peptide-78; FGF: fibroblast growth factor; G-CSF: granulocyte colony-stimulating factor; GM-CSF: granulocyte-macrophage colony-stimulating factor; GRO: growth-regulated oncogene; HGF: hepatocyte growth factor; IFN: interferon; IL: interleukin; IP: interferon-ɣ-induced protein; I-TAC: interferon-inducible T cell α-chemoattractant; LIF: leukemia inhibitory factor; MCP: monocyte chemoattractant protein; M-CSF: macrophage colony-stimulating factor; MDC: macrophage-derived chemokine; MIF: macrophage migration inhibitory factor; MIG: monokine induced by interferon-ɣ; MIP: macrophage inflammatory protein; MMP: matrix metalloproteinase; MOGAD: myelin oligodendrocyte glycoprotein associated disease; MS: multiple sclerosis; NGF: nerve growth factor; NMDAR: N-methyl-D-aspartate receptor; NMOSD: neuromyelitis optica spectrum disorder; NPSLE: neuropsychiatric syndromes of systemic lupus erythematosus; SCF: stem cell factor; SDF: stromal cell-derived factor; TBE: tick-borne encephalitis; TNF: tumor necrosis factor; TRAIL: TNF-related apoptosis-inducing ligand; TSLP: thymic stromal lymphopoietin; TWEAK: tumor necrosis factor-like weak inducer of apoptosis; VEGF: vascular endothelial growth factor

Supplementary references

1. Perdomo-Celis F, Torres MA, Ostos H, Gutierrez-Achury J, Molano V, Durán LF et al. Patterns of Local and Systemic Cytokines in Bacterial Meningitis and its Relation with Severity and Long-Term Sequelae. Biomark Insights 2015; 10:125–31.

2. Ichinose K, Arima K, Ushigusa T, Nishino A, Nakashima Y, Suzuki T et al. Distinguishing the cerebrospinal fluid cytokine profile in neuropsychiatric systemic lupus erythematosus from other autoimmune neurological diseases. Clin Immunol 2015; 157(2):114–20.

3. Kaneko K, Sato DK, Nakashima I, Ogawa R, Akaishi T, Takai Y et al. CSF cytokine profile in MOG-IgG+ neurological disease is similar to AQP4-IgG+ NMOSD but distinct from MS: a cross-sectional study and potential therapeutic implications. J Neurol Neurosurg Psychiatry 2018; 89(9):927–36.

4. Kothur K, Wienholt L, Mohammad SS, Tantsis EM, Pillai S, Britton PN et al. Utility of CSF Cytokine/Chemokines as Markers of Active Intrathecal Inflammation: Comparison of Demyelinating, Anti-NMDAR and Enteroviral Encephalitis. PLoS One 2016; 11(8):e0161656.

5. Liu J, Liu L, Kang W, Peng G, Di Yu, Ma Q et al. Cytokines/Chemokines: Potential Biomarkers for Non-paraneoplastic Anti-N-Methyl-D-Aspartate Receptor Encephalitis. Front Neurol 2020; 11:582296.

6. Bogovič P, Lusa L, Korva M, Lotrič-Furlan S, Resman-Rus K, Pavletič M et al. Inflammatory Immune Responses in Patients with Tick-Borne Encephalitis: Dynamics and Association with the Outcome of the Disease. Microorganisms 2019; 7(11).

7. Xu J, Jiang J, Zhang Y, Li W. Cytokine characteristic of cerebrospinal fluid from children with enteroviral meningitis compared to bacterial meningitis. J Clin Lab Anal 2020; 34(5):e23198.

8. Bogovič P, Kastrin A, Lotrič-Furlan S, Ogrinc K, Avšič Županc T, Korva M et al. Comparison of laboratory and immune characteristics of the initial and second phase of tick-borne encephalitis. Emerg Microbes Infect 2022; 11(1):1647–56.

9. Lee KY, Seol JH, Yi CH, Lee WH. Cerebrospinal fluid type I interferon and cytokine profiles in enteroviral meningitis according to the presence or absence of pleocytosis. Pediatr Neonatol 2021; 62(3):305–11.

10. Ye Q, Shao W-X, Shang S-Q, Shen H-Q, Chen X-J, Tang Y-M et al. Clinical Value of Assessing Cytokine Levels for the Differential Diagnosis of Bacterial Meningitis in a Pediatric Population. Medicine (Baltimore) 2016; 95(13):e3222.

11. Jafari M, Mohammadzadeh Jahani P, Choopanizadeh M, Jamalidoost M, Pourabbas B, Pouladfar G et al. Investigating the role of T helper related cytokines in cerebrospinal fluid for the differential diagnosis of bacterial meningitis in pre-treated paediatric patients. Biomarkers 2020; 25(2):171–8.

12. Byun J-I, Lee S-T, Moon J, Jung K-H, Sunwoo J-S, Lim J-A et al. Distinct intrathecal interleukin-17/interleukin-6 activation in anti-N-methyl-d-aspartate receptor encephalitis. J Neuroimmunol 2016; 297:141–7.

13. Palus M, Formanová P, Salát J, Žampachová E, Elsterová J, Růžek D. Analysis of serum levels of cytokines, chemokines, growth factors, and monoamine neurotransmitters in patients with tick-borne encephalitis: identification of novel inflammatory markers with implications for pathogenesis. Journal of Medical Virology 2015; 87(5):885–92.

14. Ygberg S, Fowler Å, Wickström R. Cytokine and Chemokine Expression in CSF May Differentiate Viral and Autoimmune NMDAR Encephalitis in Children. J Child Neurol 2016; 31(13):1450–6.

15. Kwon J-S, Park JH, Kim JY, Cha HH, Kim M-J, Chong YP et al. Diagnostic Usefulness of Cytokine and Chemokine Levels in the Cerebrospinal Fluid of Patients with Suspected Tuberculous Meningitis. Am J Trop Med Hyg 2019; 101(2):343–9.

16. Zheng K, Zhu L, Ding Y, Zhang X, Chen N, Liu G et al. Serum cytokine profile of pediatric patients with laboratory confirmed pneumococcal meningitis. J Infect Public Health 2021; 14(4):514–20.

17. Yoshio T, Okamoto H, Kurasawa K, Dei Y, Hirohata S, Minota S. IL-6, IL-8, IP-10, MCP-1 and G-CSF are significantly increased in cerebrospinal fluid but not in sera of patients with central neuropsychiatric lupus erythematosus. Lupus 2016; 25(9):997–1003.

18. Hofer LS, Mariotto S, Wurth S, Ferrari S, Mancinelli CR, Delogu R et al. Distinct serum and cerebrospinal fluid cytokine and chemokine profiles in autoantibody-associated demyelinating diseases. Mult Scler J Exp Transl Clin 2019; 5(2):2055217319848463.

19. Hirohata S, Kikuchi H. Role of Serum IL-6 in Neuropsychiatric Systemic lupus Erythematosus. ACR Open Rheumatol 2021; 3(1):42–9.

20. Srinivasan L, Kilpatrick L, Shah SS, Abbasi S, Harris MC. Elevations of novel cytokines in bacterial meningitis in infants. PLoS One 2018; 13(2):e0181449.

21. Duarte-García A, Romero-Díaz J, Juárez S, Cicero-Casarrubias A, Fragoso-Loyo H, Núñez-Alvarez C et al. Disease activity, autoantibodies, and inflammatory molecules in serum and cerebrospinal fluid of patients with Systemic Lupus Erythematosus and Cognitive Dysfunction. PLoS One 2018; 13(5):e0196487.

22. Sato E, Iikuni N, Yoshio T, Minota S, Kamatani N, Okamoto H. Soluble fractalkine in the cerebrospinal fluid of patients with neuropsychiatric lupus. Annals of the Rheumatic Diseases 2006; 65(9):1257–9.

23. Hoang TTT, Ichinose K, Morimoto S, Furukawa K, Le LHT, Kawakami A. Measurement of anti-suprabasin antibodies, multiple cytokines and chemokines as potential predictive biomarkers for neuropsychiatric systemic lupus erythematosus. Clin Immunol 2022; 237:108980.

24. Dellalibera-Joviliano R, Dos Reis ML, Cunha FdQ, Donadi EA. Kinins and cytokines in plasma and cerebrospinal fluid of patients with neuropsychiatric lupus. The Journal of Rheumatology 2003; 30(3):485–92.
